# Subjective cognitive decline and objective cognitive performance in older adults: A systematic review of longitudinal and cross‐sectional studies

**DOI:** 10.1111/jnp.12384

**Published:** 2024-07-29

**Authors:** Carl Zhou, Bassam Jeryous Fares, Kim Thériault, Brian Trinh, Morgan Joseph, Tegh Jauhal, Christine Sheppard, Patrick R. Labelle, Anjali Krishnan, Laura Rabin, Vanessa Taler

**Affiliations:** ^1^ Department of Psychiatry University of Ottawa Ottawa Ontario Canada; ^2^ Faculty of Medicine University of Ottawa Ottawa Ontario Canada; ^3^ School of Psychology University of Ottawa Ottawa Ontario Canada; ^4^ Bruyère Research Institute Ottawa Ontario Canada; ^5^ Department of Biology University of Ottawa Ottawa Ontario Canada; ^6^ Department of Psychology Carleton University Ottawa Ontario Canada; ^7^ School of Medicine New York Medical College Valhalla New York USA; ^8^ University of Ottawa Library Ottawa Ontario Canada; ^9^ Brooklyn College of the City University of New York Brooklyn New York USA

**Keywords:** ageing, cognitive function impairment, objective cognitive performance, older adults, self‐perceived cognitive functioning, subjective cognitive decline

## Abstract

Older adults with subjective cognitive decline (SCD) have a higher risk of developing future cognitive decline than those without SCD. However, the association between SCD and objective cognitive performance remains unclear. This PRISMA 2020‐compliant systematic review aims to provide a qualitative assessment of the longitudinal and cross‐sectional relationship between SCD and objective cognitive performance in different cognitive domains, in neuropsychologically healthy, community‐dwelling older adults (average age of 55 or older). To identify pertinent studies, a comprehensive search was conducted from seven databases. The National Heart, Lung and Blood Institute Quality Assessment Tool for Observational Cohort and Cross‐Sectional Studies was used to assess the quality of included studies. Inclusion criteria were met by 167 studies, which were full‐text and published between 1 January 1982 and 16 May 2023 (inclusive) in the languages of English, French, or Spanish and presenting data on objective cognitive performance in older adults with SCD. Overall, we found that SCD was associated with poorer objective cognitive performance on measures of global cognition and memory longitudinally compared to non‐SCD status, but this association was inconsistent in cross‐sectional studies. This association became stronger with the use of continuous measures of SCD as opposed to dichotomous measures. Additionally, results highlight the known lack of consistency in SCD assessment among studies and comparatively small number of longitudinal studies in SCD research.

## INTRODUCTION

Older adults with subjective cognitive decline (SCD) perceive a persistent decline in cognitive capacity while showing normal demographically‐adjusted performance on standardized neuropsychological tests (Jessen et al., 2014). Generally, people with SCD are considered cognitively healthy in clinical practice (Jessen et al., 2020). However, older adults with SCD are approximately twice as likely to develop mild cognitive impairment (MCI) or dementia as those without SCD (Wang, Wang, et al., 2021). Moreover, self‐perceived decline in cognitive functioning is one of the earliest observable signs of dementia (Jonker et al., 2000), and subjective cognitive concerns are a key diagnostic criterion for some widely used definitions of MCI (Chang et al., 2023; Petersen et al., 2001).

As the population ages and brain health awareness grows, self‐perceived declines in cognitive functioning by otherwise healthy older adults will likely become more common (Jessen et al., 2020). Although SCD can progress to cognitive disorders such as MCI and dementia (Wang, Wang, et al., 2021), few studies have directly examined the relationship between SCD and major domains of objective cognitive performance (e.g., memory, executive function, attention), particularly longitudinally. Determining the cognitive domains in which an individual is subjectively affected may help identify the potential cause of their SCD (Jessen et al., 2020), such as an increased likelihood of Alzheimer's disease (AD) among people with self‐perceived memory problems (Jessen et al., 2014). Recent studies have identified associations between subjective memory decline and subtle deficits in spatial orientation, verbal memory, and executive function, in conjunction with non‐specific structural and physiologic neural changes (Chen et al., 2021; Wang, Rao, et al., 2021). These findings suggest that domain‐specific objective cognitive performance and SCD might have a common underlying physiological mechanism. A deeper understanding of this relationship may have implications for clinical diagnosis and provide insight into the underlying disorder(s).

Cross‐sectional studies examining the relationship between memory concerns and memory impairment have produced varied results. One early review found that, although memory concerns were related to memory impairment, this link largely depended on certain participant characteristics (Jonker et al., 2000). Specifically, a stronger positive association between memory concerns and impairment was more likely in hospital‐based participants and people aged >70 compared to those with other characteristics (e.g., self‐referral, younger age). Furthermore, studies that found positive associations tended to suffer from methodological limitations, while those that did not find meaningful associations had more stringent methodologies (Reid & MacLullich, 2006). These limitations included the use of unvalidated assessments of subjective memory decline, limited assessment of objective cognitive function, and the lack of consideration for confounders such as depression and personality variables. A meta‐analysis revealed a small but significant positive correlation between subjective cognitive concerns and objective cognitive decline (Burmester et al., 2016). Although the researchers controlled for certain methodological limitations that affected previous meta‐analyses, there remained the issues of heterogeneity of included studies, potential publication bias, and the use of unvalidated assessments for SCD and objective cognitive function.

In longitudinal studies, a positive association between baseline memory concerns and cognitive decline was found (Jonker et al., 2000; Reid & MacLullich, 2006). However, methodological limitations such as non‐validated assessments of concerns and a relative lack of consideration for confounding variables remained as issues (Reid & MacLullich, 2006). Furthermore, no previous review has investigated the longitudinal relationship between non‐memory cognitive domains and cognitive performance.

Additionally, terminology is a major issue in SCD research. Although *cognitive* can refer to any cognitive domain, Rabin et al. (2015) noted that many studies, including past systematic reviews and meta‐analyses, have exclusively used memory‐related items to determine SCD status (Crumley et al., 2014; Jonker et al., 2000; Reid & MacLullich, 2006). Given that amnestic MCI patients are at a higher risk for developing AD than non‐amnestic MCI patients (Oltra‐Cucarella et al., 2018), it is not surprising that many self‐perceived cognitive functioning questionnaires are memory‐focused. However, memory questions alone are not recommended in SCD assessment (Molinuevo et al., 2017; Rabin et al., 2015); rather, measures assessing various cognitive domains of concern are favoured (Jessen et al., 2020).

Although one previous review examined the relationship between SCD and specific domains of objective cognitive performance in cross‐sectional studies (Burmester et al., 2016), no systematic review to date has examined this topic both cross‐sectionally and longitudinally. This distinction in study design is important because cross‐sectional self‐perceived cognitive functioning and objective cognitive performance are usually poorly correlated; self‐perceived decline in cognitive functioning develops over time, whereas test performance represents cognitive function at a single time point, weakening the association (Jessen, 2014).

Inherent challenges in measuring self‐perceived cognitive decline arise from the discrepancy between subjective reports of cognitive decline and objective performance measures on standardized neuropsychological tests (Crumley et al., 2014). While individuals may self‐perceive cognitive decline, their performance remains within normal limits, posing a challenge to clinicians and researchers seeking to diagnose and provide feedback about current and future functioning. Furthermore, the absence of a standardized instrument to measure self‐perceived cognitive function heightens this challenge (Rabin et al., 2020). Currently, the assessment of SCD requires both self‐perception of cognitive decline and normal performance on cognitive testing (Jessen et al., 2014); however, opinions differ regarding the optimal approach to measuring “self‐perception”. Although most studies assess SCD dichotomously, continuous variables may better capture features such as frequency and severity of cognitive concerns (Molinuevo et al., 2017). No previous review has examined the use of dichotomous versus continuous measurements in examining the relationship between SCD and objective cognitive performance.

### Goals of the current review

The current review provides a qualitative assessment of the relationship between older adults with SCD and objective cognitive performance across various domains. We summarize the literature since the establishment of SCD as a concept in 1982 (Reisberg et al., 1982). In the current study, we review various domains of self‐perceived cognitive functioning, include both cross‐sectional and longitudinal studies, and compare the outcomes of continuous versus dichotomous measurements of SCD. Our study fills gaps left by past reviews, which were largely concerned with subjective memory decline or chiefly examined cross‐sectional studies. We also consider major confounds in the relationship between SCD and cognitive performance such as depressive symptoms (i.e., sub‐threshold scores on depression scales but no formal diagnosis of mood disorder) and methodological differences.

## MATERIALS AND METHODS

We followed the Preferred Reporting Items for Systematic Reviews and Meta‐Analyses (PRISMA) checklist (Page et al., 2021) to devise our protocol, which we registered with the International Prospective Register of Systematic Reviews (CRD42020200181) before beginning the review.

### Search methods for identification of studies

A social sciences research librarian with experience in planning systematic reviews (PL) drafted, developed, and implemented a search strategy to locate pertinent published studies in AgeLine (EBSCOhost), APA PsycInfo (Ovid), CINAHL (EBSCOhost), Embase (Ovid), MEDLINE (Ovid), Scopus, and Web of Science. The final search strategy included relevant database‐specific subject headings and keywords for both concepts of the review and was executed on 16 May 2023. No limits or restrictions were used in any of the database searches (the complete search strategy is available in Appendix S1). We imported citations into Covidence (Veritas Health Innovation Ltd., Melbourne, Australia). Duplicate references were identified and removed once imported into Covidence. Additional duplicates were identified and excluded while screening references.

### Selection criteria

All included articles: (1) were peer‐reviewed, longitudinal, or cross‐sectional studies published between 1 January 1982 and 16 May 2023 (inclusive) in the languages of English, French, or Spanish; (2) involved community‐dwelling older adult participants (average age of 55+) with SCD; (3) involved participants without concurrent MCI, prodromal AD, dementia, psychiatric, neurological, or substance use disorders, or medication use that could explain cognitive decline (e.g., anticholinergics and benzodiazepines; Koyama et al., 2014); (4) assessed objective cognitive performance using at least one test of cognitive function against non‐SCD controls; and (5) explored the association between SCD and objective cognitive performance (significant correlation not required). These criteria were selected based on the SCD conceptual framework for research created by Jessen et al. (2014). An average age of 55+ years was chosen following many studies on ageing and subjective cognitive decline (Mol et al., 2006; Morrison & Oliver, 2023; Verfaillie et al., 2018). Our search was not limited to specific cognitive domains, and domains assessed objectively included global cognition, memory, executive functions, attention, etc. Please refer to Tables 1 and 2 for an exhaustive list of cognitive domains included in this review.

**TABLE 1:** Objective cognitive results of cross‐sectional studies.

Cognitive domain	Method of SCD assessment									
	Medical help‐seekers		Self‐reported dichotomous measure		Worry‐endorsed dichotomous measure		Informant‐endorsed dichotomous measure		Continuous measure	
	Significant difference^a^	No difference	Significant difference	No difference	Significant difference	No difference	Significant difference	No difference	Significant correlation^b^	No correlation
Global cognition	7	29	31	65	1	15	5	7	14	11
Global cognition (MMSE not included)	4	8	18	17	1	8	3	3	9	2
Premorbid Intelligence	1	4^c^	0	9^c^	0	3	0	1	2	1
Memory	7	26	31	65^c^	3	17	6	3	31	18
Prospective memory	1	1	3	2^c^	0	1	0	0	0	0
Retrospective memory	0	2	1	0	0	0	0	0	0	0
Attention	2	14	2	42	0	9	0	5	7	14
Language	4	11	16	49	2	5	6	3	10	12
Executive functions	4	27	20	69^c^	2	23	2	5	14	22
Visuo‐spatial	0	9	2	19	0	2	1	1	3	4
Reasoning	0	1	0	1	0	0	0	0	0	0
Praxis functioning	0	0	0	1	0	0	0	0	0	0
Spatial navigation	0	0	0	1	0	2	0	0	0	0
Decision‐making	0	0	0	0	0	1	0	0	0	0
Spatiotemporal context of vision for action	0	0	0	1	0	0	0	0	1	0
Psychomotor speed	0	0	1	0	0	0	0	0	1	0
Visual processing	0	0	0	0	0	0	0	0	0	1
Visual perception	0	0	0	1	0	0	0	0	0	3
Orientation	0	0	0	2	0	0	0	1	1	0
Processing speed	3	2	3	9	0	0	2	0	0	1

Abbreviations: MMSE, Mini‐Mental State Examination; SCD, Subjective cognitive decline.

^a^ Number of measures indicating a better performance by non‐SCD controls than SCD participants.

^b^ Number of measures indicating significant negative correlations with continuous measures of SCD.

^c^ Indicates that one study observed a better performance by SCD participants.

**TABLE 2:** Objective cognitive results of longitudinal studies.

Cognitive domain	Method of SCD assessment							
	Self‐reported dichotomous measure		Continuous measure		Worry‐endorsed dichotomous measure		Informant‐endorsed dichotomous measure	
	Significant difference^a^	No difference	Significant correlation^b^	No correlation	Significant difference^a^	No difference	Significant difference^a^	No difference
Global cognition	5	1	3	1	1	0	1	0
Memory	13	7	1	1	1	0	0	1
Language	1	9	0	0	0	1	2	1
Attention	1	5	1	0	0	0	0	1
Executive functioning	3	7	0	0	0	1	1	1
Processing speed	2	3	0	0	0	0	2	0
Psychomotor speed	1	0	1	0	0	0	0	0
Visuo‐spatial	2	1	0	0	0	0	0	0

Abbreviation: SCD, subjective cognitive decline.

^a^ Number of measures indicating a better performance by non‐SCD controls than SCD participants.

^b^ Number of measures indicating significant negative correlations with continuous measures of SCD.

### SCD operationalization

SCD status was determined based on the original research criteria from Jessen et al. (2014): a self‐perceived decline in cognitive function in the context of normal neuropsychological performance, where the decline was not the result of physical or psychiatric illness that could affect cognitive ability. Following Molinuevo et al. (2017), we included studies where concerns about cognitive functioning were self‐ or informant‐reported, or both self‐ and informant‐reported.

### Study selection and data extraction

Reference screening, data extraction, and risk of bias assessment were conducted by six reviewers; during each phase, two reviewers independently conducted these steps, and a third reviewer resolved any disagreement. We extracted information about study design (longitudinal/cross‐sectional), follow‐up period, inclusion and exclusion criteria, demographic information, recruitment method, SCD variable (dichotomous/continuous), cognitive domains and measures used, findings, and limitations (see Appendix S3 for details).

### Quality assessment

We assessed study quality using the National Heart, Lung and Blood Institute Quality Assessment Tool for Observational Cohort and Cross‐Sectional Studies (NHLBI, 2014). Each study received a score out of 100%. Following Mogre et al. (2019), we considered a score of >80% (≥12/14) good quality; 60%–80% (9/14 to 11/14) fair quality; and <60% (≤8/14) low quality. A very low‐quality category was added to describe studies with a score of <40%. Appendix S2 presents the quality assessment consensus outcome.

## RESULTS

**FIGURE 1:**
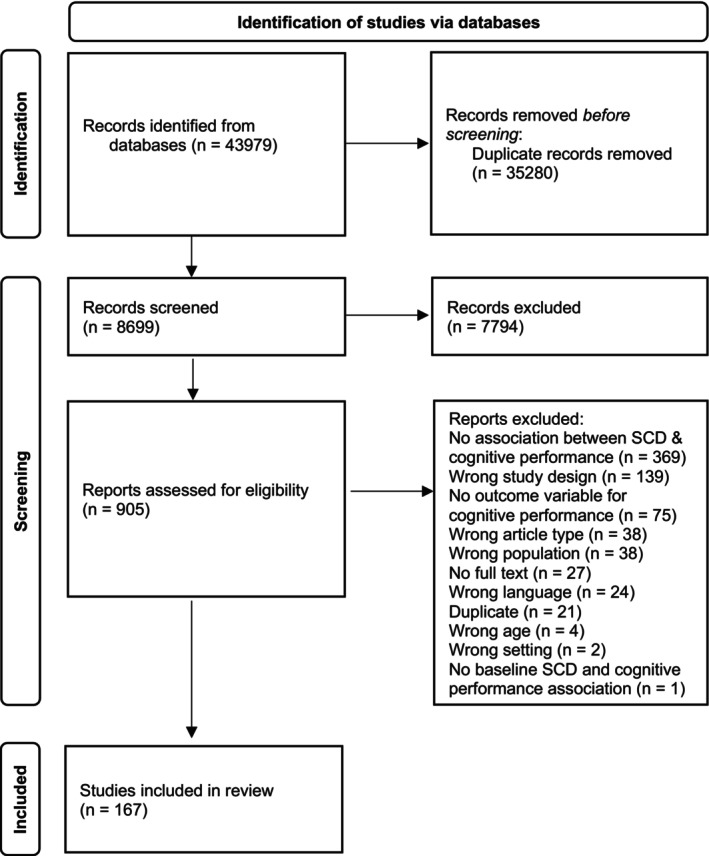
Study selection process in accordance with the Preferred Reporting Items for Systematic Reviews and Meta‐Analyses 2020 (Page et al., 2021). SCD, subjective cognitive decline.

The initial search yielded 43,979 publications: 35,280 duplicate publications were removed, and 7794 publications were excluded following title and abstract screening. Of the remaining 905 full‐text articles, 738 did not meet the inclusion criteria, resulting in 167 articles (Figure 1).

### Study characteristics

#### Study design and setting

167 publications between the years of 1994 and 2023 were identified (see Appendix S3 for details). Most studies were conducted in the United States (*n* = 35), followed by Australia (*n* = 14). The remaining studies were conducted throughout Europe, Asia, North America, and South America, and sometimes involved multiple locations. 87 included memory clinic patients, 19 drew from the general population, and 61 combined these recruitment methods. We identified 128 self‐designated cross‐sectional studies and 39 self‐designated longitudinal studies, with a mean follow‐up time of 2.17 years (*SD* = 1.64 years). The mean sample size across all studies was 459.53 participants (*SD* = 1550.60).

#### Study findings

Self‐perceived cognitive functioning was assessed via questionnaires (*n* = 79; 47.3%), interviews (*n* = 67; 40.1%), and other formats such as a single question, clinical assessment, or a combination of modalities (*n* = 21; 12.6%). Most studies assessed SCD dichotomously (*n* = 126; 75.4%), with a single question relating to subjective memory or cognitive concerns. Some studies required SCD participants to report worry about their SCD or to have an informant confirm their SCD status. When long‐form questionnaires and interviews were used, SCD was typically assessed using a scale and determined by a cut‐off score (*n* = 31; 18.6%).

The SCD domains included memory (*n* = 116), global cognition (*n* = 56), or others (e.g., attention, language, visuo‐spatial abilities, executive function) (*n* = 8). Ten studies assessed multiple domains. To assess objective cognitive performance, 110 unique neuropsychological tests were used, of which 48 were used only once (see Appendix S4). The most common neuropsychological tests assessed global cognition (Mini‐Mental State Examination, used in 120 studies), attention (Trail‐Making Test Parts A and B, used in 76 studies), confrontation naming (Boston Naming Test, used in 37 studies), working memory (Digit Span Forward and Backward, used in 37 studies), and verbal memory (Rey Auditory Verbal Learning Test, used in 32 studies) (see Appendix S3 for an overview of each study's characteristics, including neuropsychological measures).

#### Cross‐sectional studies

Inconsistent findings were observed with respect to associations between SCD and objective cognitive test scores in cross‐sectional studies. Dichotomous assessments of SCD included self‐report, worry‐endorsed, and informant‐endorsed dichotomous measures. Across studies, no group differences in global cognition were found for 87 cognitive measures, while non‐SCD participants outperformed SCD participants on 37 measures. Further, the MMSE accounted for many measures of global cognition across studies. When excluding the MMSE, which is not considered to be sensitive to MCI (Hoops et al., 2009; Nasreddine et al., 2005), non‐SCD participants outperformed SCD participants on 22 measures, while no group differences were observed on 28 measures of global cognition (Table 1).

For memory performance specifically, 84 measures indicated no group differences, 40 measures showed lower performance in the SCD group, and one measure indicated a better performance in the SCD group (Table 1). Most studies found no significant group differences in language, executive functioning, and attention (Table 1).

Few studies compared self‐ and informant‐reported SCD, demonstrating conflicting findings. Informant‐reported concerns correlated with worse performance on six measures of memory and six measures of language, while no correlation was observed for three measures of memory and three measures of language. Conflicting results were also present across other cognitive domains (see Table 1). One study found that concurrent self‐ and informant‐reported concerns predicted worse performance in global cognition and memory, although results were mixed for language and information processing, and no effects were found for attention (Gifford et al., 2014). These findings demonstrate the potential importance of informant reports of cognitive concerns.

Studies using a continuous measurement of SCD found significant negative correlations between SCD severity and memory performance for 31 measures, while no correlation was found for 18 measures. Conflicting findings were also reported for attention, executive functioning, and language (see Table 1).

#### Longitudinal studies

The findings with respect to SCD and objective cognitive decline were more consistent in longitudinal studies. Most studies found that the SCD group was more likely to decline at follow‐up compared to the non‐SCD group on measures of global cognition and memory (see Table 2), while group differences were generally not observed at follow‐up in other domains such as executive function, language, and attention.

However, some nuances in study findings also emerged. For example, some studies reported more decline in SCD participants in specific cognitive domains other than global cognition and memory at follow‐up compared to non‐SCD participants (e.g., immediate and delayed recall, Koppara et al., 2015; semantic fluency and executive function, Rosas et al., 2022; psychomotor speed, Kielb et al., 2017). Similarly, greater improvement at follow‐up in non‐SCD compared to SCD participants has been observed in immediate and delayed recall (Kielb et al., 2017; Verfaillie et al., 2018). One study found baseline differences in general information processing and delayed recall between SCD and non‐SCD control groups, but these differences were not present at annual follow‐up (Mol et al., 2006). However, other research has shown that SCD participants experience greater rates of decline across multiple cognitive domains, including global cognition, episodic memory, semantic memory, perceptual speed, working memory, and visuo‐spatial abilities (Morrison & Oliver, 2023).

With respect to the role of informant report, Gifford et al. (2014) found that participants with both self‐ and informant‐reported cognitive concerns showed greater declines in global cognition, memory, attention, and executive function compared to non‐SCD controls, while participants with only informant‐reported concerns performed worse at follow‐up on measures of processing speed, working memory, and visuo‐spatial processing compared to non‐SCD controls. Interestingly, those with self‐ but not informant‐reported concerns only differed from non‐SCD controls in memory measures. In interpreting these findings, it is important to consider the role of practice effects, because the same cognitive tests were used at baseline and follow‐up in several studies (Kielb et al., 2017; Mol et al., 2006; Morrison & Oliver, 2023; Verfaillie et al., 2018).

Studies using a continuous measure of SCD more consistently found associations between SCD and subsequent cognitive trajectory compared to studies using a dichotomous measure (Amariglio et al., 2018; Dufouil et al., 2005; Lehrner et al., 2016). Slavin et al. (2015) reported that multi‐domain SCD did not predict objective cognitive decline, but memory‐specific items did. In contrast, only one study (Buckley et al., 2016) did not detect a significant association between subjective memory decline and objective episodic memory decline. However, this study did not measure domains of subjective and objective cognition beyond memory. These findings demonstrate the potential strength of using continuous measures of SCD over dichotomous measures in assessing objective cognitive decline.

### Depressive symptoms

A total of 93 studies investigated the associations between depressive symptoms and SCD longitudinally (*n* = 14) and cross‐sectionally (*n* = 79). In cross‐sectional research, 54 studies reported that SCD was significantly associated with depressive symptoms, while 25 studies found no significant differences. Among those studies reporting a significant effect, the majority reported higher depression scores in all groups (SCD, MCI, or AD) in comparison with the non‐SCD group. In longitudinal research, 12 studies found that depressive symptoms at follow‐up were significantly associated with SCD at follow‐up, with only two reporting that SCD was independent of depressive symptoms. Generally, the findings with respect to SCD and depressive symptoms were more consistent in longitudinal compared to cross‐sectional studies. These results suggest an association between SCD and depressive symptoms, although the causal nature of the relationship remains unclear.

### Quality assessment

The quality assessment results are displayed in Table 3. Around half of the included studies were considered low or very low quality. This stemmed mainly from the predominance of cross‐sectional studies, as well as the poor description of the recruitment methods (e.g., participation rates for the SCD and non‐SCD samples). Several studies also failed to provide sample size justification, power description, or variance and effect estimates. Many studies compared the cognitive scores of participants reporting SCD and non‐SCD without considering the severity and extent of SCD, especially given that only a limited number of studies used a continuous measure of self‐perceived cognitive functioning (see Table 4 for recommended methodological features for future studies based on the results of the quality assessment).

**TABLE 3:** Quality assessment: Results of National Heart, Lung and Blood Institute Quality Assessment Tool for Observational Cohort and Cross‐Sectional Studies (NHLBI, 2014).

Assessment tool question	% of studies with a “yes” answer
1. Was the research question or objective in this paper clearly stated?	100.00
2. Was the study population clearly specified and defined?	32.14
3. Was the participation rate of eligible persons at least 50%?	2.98
4. Were all the subjects selected or recruited from the same or similar populations (including the same time period)? Were inclusion and exclusion criteria for being in the study prespecified and applied uniformly to all participants?	60.71
5. Was a sample size justification, power description, or variance and effect estimates provided?	36.90
6. For the analyses in this paper, were the exposure(s) of interest measured prior to the outcome(s) being measured?	16.07
7. Was the timeframe sufficient so that one could reasonably expect to see an association between exposure and outcome if it existed?	12.50
8. For exposures that can vary in amount or level, did the study examine different levels of the exposure as related to the outcome (e.g., categories of exposure, or exposure measured as continuous variable)?	24.40
9. Were the exposure measures (independent variables) clearly defined, valid, reliable, and implemented consistently across all study participants?	92.86
10. Was the exposure(s) assessed more than once over time?	10.12
11. Were the outcome measures (dependent variables) clearly defined, valid, reliable, and implemented consistently across all study participants?	99.40
12. Were the outcome assessors blinded to the exposure status of participants?	5.95
13. Was loss to follow‐up after baseline 20% or less?	7.14
14. Were key potential confounding variables measured and adjusted statistically for their impact on the relationship between exposure(s) and outcome(s)?	70.24
Average study quality total score (*SD*)	40.82 (12.70%)

**TABLE 4:** Recommended methodological features for future studies.

Recommendation	Description
Longitudinal design	Considering SCD's potential predictive value for objective cognitive decline and development of dementia (see Table 3; Burmester et al., 2016; Crumley et al., 2014; Jonker et al., 2000; Reid & MacLullich, 2006), future studies should implement a longitudinal design with reasonably sufficient follow‐up period to assess the correlation between SCD and decline on objective assessments of cognition.
Assessing SCD as a continuous variable	A continuous assessment of SCD may be more sensitive to objective cognitive decline than a dichotomous measure (see Tables 3 and 4). Future studies may benefit from measuring SCD continuously and investigating the association between the severity of SCD and objective measures of cognition. However, the current review cannot make a recommendation on which continuous measure of SCD to use due to lack of consistency across these measures in the studies under review (see Morrison et al., 2022).
Comprehensive description of control and SCD samples	Results varied across studies depending on control and SCD samples recruited (see Tables 3 and 4). A comprehensive description of the samples is necessary to allow for accurate comparisons between studies. Further, there is value in including clinical and non‐clinical SCD samples for better assessment of the nature of SCD and its correlation to objective cognition and cognitive decline.
Controlling for confounding variables	The current review highlights the importance of controlling for confounding variables that can influence cognitive performance such as age, education, gender, anxiety, and depression (see Results). This is instrumental to understanding the extent of the association between objective cognitive performance and SCD.

## DISCUSSION

Our examination of data from 167 ageing studies yielded mixed results for the association between SCD and objective cognitive performance. Overall, the association between SCD and cognitive performance was stronger in longitudinal than cross‐sectional studies, consistent with Reid and MacLullich (2006), who found that longitudinal memory concern was associated with objective cognitive decline, while cross‐sectional memory concern may be indicative of depression or neuroticism in some cases. This finding is unsurprising because SCD has construct validity in predicting future objective decline (Wang, Wang, et al., 2021). Furthermore, at a conceptual level, SCD refers to the longitudinal nature of a person's cognitive trajectory, rather than their cross‐sectional cognitive abilities (Koppara et al., 2015). In fact, the establishment of cognitive normality on standardized testing is a prerequisite for SCD classification (Jessen et al., 2014). Therefore, any relationship between SCD and objective cognition should be more difficult to detect cross‐sectionally than longitudinally.

A novel finding was that the association between SCD and objective cognition was stronger in studies using continuous rather than dichotomous measurements of SCD, likely because continuous scales better capture the severity or degree of change, while dichotomous scales are more useful for group classification purposes. This finding reinforces Molinuevo et al.'s (2017) suggestion that researchers should consider using continuous variables in SCD assessment, particularly since SCD is likely not dichotomous in nature. Furthermore, older adults may perceive dichotomous scales as being restrictive, which can affect the accuracy of an assessment's results (Carp, 1989).

Generally, we did not find domain‐specific associations between SCD measurement and objective cognitive performance. Prior research suggests that subjective memory decline predicts future AD (Jessen et al., 2014), and subjective declines in temporal orientation, language, and executive function may be associated with preclinical AD (La Joie et al., 2016; Valech et al., 2018). The studies included in the present review used a variety of cognitive measures, but this finding is not necessarily a limitation. If the assessments are clearly defined, valid, and reliable, their heterogeneity could provide more nuanced insight into the various domains of cognitive function and how they are affected by SCD. Specialized cognitive tests are generally designed to detect subtle cognitive deficits, while standardized general cognition tests have a relatively wide range of what is considered cognitively normal (The National Academies, 2015).

We found an association between depressive symptoms and SCD in both longitudinal and cross‐sectional studies, congruent with past reviews (Burmester et al., 2016; Reid & MacLullich, 2006). There are several possible interpretations of this relationship. Depressive symptoms may be the primary cause of SCD because depression can lead to poorer cognitive performance, which in turn may trigger SCD (Markova et al., 2019). Depressive symptoms can also interfere with self‐perception of cognitive performance (Lubitz et al., 2020; Markova et al., 2019). For example, depression could contribute to a negative assessment of one's own memory performance, which would lead to memory concerns (Chin et al., 2014). However, in these cases, the cause could be either normal ageing or pathology (Chin et al., 2014). Depression could also be triggered by awareness of cognitive decline (Markova et al., 2019). While the causal relationship between depressive symptoms and SCD is difficult to determine, the present findings clearly demonstrate the importance of controlling for depressive symptoms when assessing SCD (Dufouil et al., 2005).

### Limitations

Some caution should be taken when drawing conclusions from the current review due to the observed sampling bias, heterogeneity, and lack of consistency in recruitment and assessment between studies. An important limitation is the lack of consensus regarding SCD assessment. Many studies have focused on subjective memory concerns or decline, rather than exploring a wider range of cognitive domains. When examining subjective cognitive or memory decline, there was also inconsistency about the timeframe of comparison for decline. Participants were asked to report subjective decline relative to a certain point in the past, which varied across studies. SCD assessments also varied considerably, including whether the assessment was dichotomous or continuous, as well as the specific measures used.

An important point relates to the presence of self‐ versus informant‐reported decline. Informant‐reported decline may better predict objective cognitive performance and progression to dementia than self‐reported SCD alone, although informant report also has limitations related to factors such as type and quality of relationship and frequency of contact (Chang et al., 2023).

Caution must be taken when generalizing these findings. Many studies (*n* = 55 of 167) recruited SCD participants from memory clinics, creating a source of sample bias, because SCD help‐seekers might differ from the general population (Rami et al., 2014). Some studies also reported a lack of generalizability due to factors such as race/ethnicity, gender, and region. Most of the studies were cross‐sectional, and many had relatively small sample sizes. Large longitudinal studies are required to better assess the interaction between SCD and objective cognitive decline. Lastly, several studies noted limitations relating to a lack of assessment of affect (i.e., emotional expression), AD biomarkers, beliefs and fear of developing dementia, or family history of dementia. These factors might affect SCD development, and their incorporation into future studies might contribute to a better predictive tool for objective cognitive performance. Finally, our study was qualitative, and future reviews should consider quantitative approaches, to establish a better understanding of the correlation between SCD and objective cognitive function and decline. However, we recognize that this may be difficult given the heterogeneity between studies, as described above, and the lack of validated SCD assessments.

### Suggestions for future research

We agree with Rabin et al. (2015) and Burmester et al. (2016) that there are widespread inconsistencies and a lack of validation among SCD assessments used in SCD research. Although a gold standard SCD assessment is not yet available, Molinuevo et al. (2017) recommended a number of criteria that should be considered in any SCD study to enhance generalizability and comparability across studies: (1) inclusion of self‐ or informant‐report data based on the appropriateness for the study sample and setting, (2) use of validated SCD assessments that are suitable for the target population and research question, (3) the appropriateness of comparison groups and timeframes of comparison, and (4) the use of cut‐offs to determine SCD status, with explanations of how cut‐offs were derived. Based on the evidence from this review, we propose an additional criterion: the use of continuous measures in SCD assessment should be favoured over dichotomous measures when the purpose is to identify individuals at risk of future decline. We do not have data‐driven recommendations regarding domain‐specific measurements of SCD or objective cognitive decline, because the evidence regarding these methods is currently insufficient to draw conclusions. However, it seems reasonable to include multiple cognitive domains as part of any comprehensive assessment.

Looking forward, the Subjective Cognitive Decline Initiative (SCD‐I), a working group of SCD researchers, has prioritized the creation of a validated SCD scale that harmonizes existing measures (Rabin et al., 2015, 2023). In their recent study, the working group successfully harmonized 601 items from self‐perceived cognitive functioning questionnaires used in multiple international studies (Rabin et al., 2023). They found that items capturing specific problems with memory, executive functioning, attention, language, calculation, and visuo‐spatial skills were the most psychometrically robust for measuring self‐perceived cognitive functioning. However, validity testing of items must occur before a reliable SCD questionnaire can be developed and broadly disseminated. For the time being, we recommend that researchers ensure clarity by selecting their SCD assessments based on existing guidelines, and clearly stating the rationale for their chosen instrument(s).

The incorporation of mood assessments is critical in SCD research. Depression and other mood‐related variables should be assessed to exclude individuals with a psychiatric diagnosis (as per the SCD research criteria developed by Jessen et al., 2014), and to include those with sub‐threshold symptoms in the study sample. Hill et al. (2016) found, in a systematic review, that SCD is often positively associated with symptoms of depression. However, the causal relationship is unclear. Thus, it is recommended that these participants be included, and their symptoms assessed and accounted for in statistical models when appropriate (Jessen et al., 2014). While this recommendation is not new, we note that in this review, depressive symptoms were only reported by 93 of 167 total studies.

We encourage SCD researchers to pursue more and higher‐quality prospective longitudinal studies assessing the relationship between SCD and objective cognitive performance. These studies account for a small minority of our reviewed studies while showing promising results in multiple areas. Furthermore, although some evidence suggests a domain‐specific relationship between SCD and objective cognitive performance, more research is needed in this field to draw firmer conclusions. Finally, to improve the generalizability of findings, studies should also consider recruiting SCD and non‐SCD control participants from the general population rather than help‐seeking samples.

## CONCLUSIONS

Current evidence suggests that SCD is associated with objective cognitive performance longitudinally, but inconsistently associated in cross‐sectional studies. This association among longitudinal studies is strengthened when continuous rather than dichotomous measures of SCD are employed. Symptoms of depression are also associated with SCD. Future research should strictly adhere to existing guidelines (Jessen et al., 2014; Molinuevo et al., 2017) to ensure consistency, comparability, and generalizability within the SCD research field. The creation of a gold standard SCD assessment, driven by prior research results and expert opinion and shared across settings and studies, might be beneficial in furthering this goal.

## AUTHOR CONTRIBUTIONS

**Carl Zhou:** Conceptualization; investigation; writing – original draft; methodology; writing – review and editing; data curation; supervision. **Bassam Jeryous Fares:** Writing – original draft; methodology; writing – review and editing; data curation; supervision; investigation; conceptualization. **Kim Thériault:** Investigation; writing – original draft; methodology; writing – review and editing; data curation. **Brian Trinh:** Writing – original draft; methodology; writing – review and editing; data curation; investigation. **Morgan Joseph:** Writing – original draft; investigation; methodology; writing – review and editing; data curation. **Tegh Jauhal:** Investigation; writing – original draft; methodology; writing – review and editing; data curation. **Christine Sheppard:** Conceptualization; writing – review and editing; project administration; supervision; methodology. **Patrick R. Labelle:** Methodology; writing – review and editing; software. **Anjali Krishnan:** Writing – original draft; writing – review and editing; methodology; software; formal analysis. **Laura Rabin:** Methodology; writing – review and editing; project administration; supervision. **Vanessa Taler:** Methodology; writing – review and editing; project administration; supervision; investigation; conceptualization; funding acquisition.

## CONFLICT OF INTEREST STATEMENT

The authors have no conflicts of interest to declare.

## ETHICAL APPROVAL

Institutional review board approval was not needed because this study did not involve human or animal participants.

## Supporting information


Appendix S1.

Appendix S2.

Appendix S3.

Appendix S4.

Appendix S5.

Appendix S6.

## Data Availability

The data for this study are available. See Appendices S2 and S3 for data extraction results.
