# Supplementary material for: Subjective cognitive decline and objective cognitive performance in older adults: A systematic review of longitudinal and cross‐sectional studies
Source: J Neuropsychol. 2024 Jul 29;19(1):98–114. doi: 10.1111/jnp.12384 (PMC11891377; doi:10.1111/jnp.12384)
Supplement: Supplementary file 1 — Appendix S1. [file JNP-19-98-s002.docx]

**Search Strategy**

The following databases were searched on May 16, 2023:

- AgeLine (EBSCOhost) – 734 results
- APA PsycInfo (Ovid) – 1698 results
- CINAHL (EBSCOhost) – 1848 results
- Embase (Ovid) – 5680 results
- MEDLINE (Ovid) – 3713 results
- Scopus – 2034 results
- Web of Science – 4726 results
- Total (before duplicates removed) – 20433 results

The following strategies were used for each database.

AgeLine (EBSCOhost)

1. TI("subjective cognitive decline" or "subjective cognitive concern*" or "subjective cognitive complain*" or "subjective cognitive impair*" or "subjective cognitive concern*" or "subjective cognitive loss*") OR AB("subjective cognitive decline" or "subjective cognitive concern*" or "subjective cognitive complain*" or "subjective cognitive impair*" or "subjective cognitive concern*" or "subjective cognitive loss*")
2. TI("subjective memory decline" or "subjective memory concern*" or "subjective memory complain*" or "subjective memory impair*" or "subjective memory concern*" or "subjective memory loss*") OR AB("subjective memory decline" or "subjective memory concern*" or "subjective memory complain*" or "subjective memory impair*" or "subjective memory concern*" or "subjective memory loss*")
3. TI((preclinical or prodromal) N2 (alzheimer* or dementia*)) OR ((preclinical or prodromal) N2 (alzheimer* or dementia*))
4. TI("cognitive concern*" or "cognitive complaint*") OR AB("cognitive concern*" or "cognitive complaint*")
5. S1 or S2 or S3 or S4

APA PsycInfo (Ovid)

1. ("subjective cognitive decline" or "subjective cognitive concern*" or "subjective cognitive complain*" or "subjective cognitive impair*" or "subjective cognitive concern*" or "subjective cognitive loss*").ti,ab
2. ("subjective memory decline" or "subjective memory concern*" or "subjective memory complain*" or "subjective memory impair*" or "subjective memory concern*" or "subjective memory loss*").ti,ab
3. ((preclinical or prodromal) adj2 (alzheimer* or dementia*)).ti,ab
4. ("cognitive concern*" or "cognitive complaint*").ti,ab
5. or/1-4
6. aging/
7. ((old* or aging or senior* or retire* or geriatric*) adj3 (people* or person* or adult* or women* or men* or male* or female* or citizen* or residen* or population*)).ti,ab
8. (elder* or old age or aged).ti,ab
9. or/6-8
10. 5 and 9

CINAHL (EBSCOhost)

1. TI("subjective cognitive decline" or "subjective cognitive concern*" or "subjective cognitive complain*" or "subjective cognitive impair*" or "subjective cognitive concern*" or "subjective cognitive loss*") OR AB("subjective cognitive decline" or "subjective cognitive concern*" or "subjective cognitive complain*" or "subjective cognitive impair*" or "subjective cognitive concern*" or "subjective cognitive loss*")
2. TI("subjective memory decline" or "subjective memory concern*" or "subjective memory complain*" or "subjective memory impair*" or "subjective memory concern*" or "subjective memory loss*") OR AB("subjective memory decline" or "subjective memory concern*" or "subjective memory complain*" or "subjective memory impair*" or "subjective memory concern*" or "subjective memory loss*")
3. TI((preclinical or prodromal) N2 (alzheimer* or dementia*)) OR AB((preclinical or prodromal) N2 (alzheimer* or dementia*))
4. TI("cognitive concern*" or "cognitive complaint*") OR AB("cognitive concern*" or "cognitive complaint*")
5. S1 or S2 or S3 or S4
6. (MH "Aged") OR (MH "Aged, 80 and Over") OR (MH "Centenarians")
7. TI((old* or aging or senior* or retire* or geriatric*) N3 (people* or person* or adult* or women* or men* or male* or female* or citizen* or residen* or population*)) OR AB((old* or aging or senior* or retire* or geriatric*) N3 (people* or person* or adult* or women* or men* or male* or female* or citizen* or residen* or population*))
8. TI(elder* or old age or aged) OR AB(elder* or old age or aged)
9. S6 or S7 or S8
10. S5 AND S9

Embase (Ovid)

1. ("subjective cognitive decline" or "subjective cognitive concern*" or "subjective cognitive complain*" or "subjective cognitive impair*" or "subjective cognitive concern*" or "subjective cognitive loss*").ti,ab
2. ("subjective memory decline" or "subjective memory concern*" or "subjective memory complain*" or "subjective memory impair*" or "subjective memory concern*" or "subjective memory loss*").ti,ab
3. ((preclinical or prodromal) adj2 (alzheimer* or dementia*)).ti,ab
4. ("cognitive concern*" or "cognitive complaint*").ti,ab
5. or/1-4
6. exp aged/
7. ((old* or aging or senior* or retire* or geriatric*) adj3 (people* or person* or adult* or women* or men* or male* or female* or citizen* or residen* or population*)).ti,ab
8. (elder* or old age or aged).ti,ab
9. or/6-8
10. 5 and 9

MEDLINE (Ovid)

1. ("subjective cognitive decline" or "subjective cognitive concern*" or "subjective cognitive complain*" or "subjective cognitive impair*" or "subjective cognitive concern*" or "subjective cognitive loss*").ti,ab
2. ("subjective memory decline" or "subjective memory concern*" or "subjective memory complain*" or "subjective memory impair*" or "subjective memory concern*" or "subjective memory loss*").ti,ab
3. ((preclinical or prodromal) adj2 (alzheimer* or dementia*)).ti,ab
4. ("cognitive concern*" or "cognitive complaint*").ti,ab
5. or/1-4
6. exp aged/
7. ((old* or aging or senior* or retire* or geriatric*) adj3 (people* or person* or adult* or women* or men* or male* or female* or citizen* or residen* or population*)).ti,ab
8. (elder* or old age or aged).ti,ab
9. or/6-8
10. 5 and 9

Scopus

1. TITLE-ABS("subjective cognitive decline" or "subjective cognitive concern*" or "subjective cognitive complain*" or "subjective cognitive impair*" or "subjective cognitive concern*" or "subjective cognitive loss*")
2. TITLE-ABS("subjective memory decline" or "subjective memory concern*" or "subjective memory complain*" or "subjective memory impair*" or "subjective memory concern*" or "subjective memory loss*")
3. TITLE-ABS((preclinical or prodromal) W/2 (alzheimer* or dementia*))
4. TITLE-ABS("cognitive concern*" or "cognitive complaint*")
5. #1 OR #2 OR #3 OR #4
6. TITLE-ABS((old* or aging or senior* or retire* or geriatric*) W/3 (people* or person* or adult* or women* or men* or male* or female* or citizen* or residen* or population*))
7. TITLE-ABS(elder* or old age or aged)
8. #6 OR #7
9. #5 OR #8

Web of Science

1. TS=("subjective cognitive decline" or "subjective cognitive concern*" or "subjective cognitive complain*" or "subjective cognitive impair*" or "subjective cognitive concern*" or "subjective cognitive loss*")
2. TS=("subjective memory decline" or "subjective memory concern*" or "subjective memory complain*" or "subjective memory impair*" or "subjective memory concern*" or "subjective memory loss*")
3. TS=((preclinical or prodromal) NEAR/2 (alzheimer* or dementia*))
4. TS=("cognitive concern*" or "cognitive complaint*")
5. #1 OR #2 OR #3 OR #4
6. TS=((old* or aging or senior* or retire* or geriatric*) NEAR/3 (people* or person* or adult* or women* or men* or male* or female* or citizen* or residen* or population*))
7. TS=(elder* or old age or aged)
8. #6 OR #7
9. #5 AND #8

## Study Selection Process

Six reviewers participated in reference screening, data extraction, and risk of bias assessment. Title and abstract screening, full-text screening, data extraction, and risk of bias assessment were performed in Covidence (Veritas Health Innovation Ltd, Melbourne, Australia). During each of these phases, two reviewers independently screened articles, extracted data, or assessed the risk of bias for each article. A third reviewer resolved any conflicts that arose between the two reviewers. The third reviewer avoided resolving conflicts in which they themselves were involved. To obtain full-text articles that could not be found in the library database or via internet search, one reviewer contacted the first authors of those manuscripts by email.

##

## Data Extraction and Synthesis

CZ piloted the data extraction and risk of bias assessment tools. CZ provided training to the authors to perform data extraction and risk of bias assessment. We did not revise the tools during the data extraction process.

We extracted the following information from articles that were retained after full-text screening: 1) title of article, first author’s name, year of publication, and geographic location of study; 2) study design (e.g., longitudinal, cross-sectional) and follow-up period for longitudinal studies; 3) study inclusion and exclusion criteria; 4) participant demographic information (e.g., SCD and comparison sample size, age, gender, education, race); 5) recruitment method; 6) SCD variable type (e.g., continuous, dichotomous) and method of SCD assessment; 7) objective cognitive performance domains assessed and neuropsychological function tests used; 8) findings related to SCD and objective cognitive performance; 9) other relevant findings (e.g., related to depressive symptoms, demographic data, physical health, personality); and 10) study limitations according to the authors. We described and summarized our results qualitatively.
