# Supplementary material for: Subjective cognitive decline and objective cognitive performance in older adults: A systematic review of longitudinal and cross‐sectional studies
Source: J Neuropsychol. 2024 Jul 29;19(1):98–114. doi: 10.1111/jnp.12384 (PMC11891377; doi:10.1111/jnp.12384)
Supplement: Supplementary file 4 — Appendix S4. [file JNP-19-98-s001.pdf]

Cognitive Performance Tests Used Across Studies

ACE-III	4
ACE-R	3
AKT	2
ADAS	2
Animal Naming	22
BNT	41
BVRT	2
CVLT-II	16
CAMCOG	4
CAMCOG-R	3
CCRT	2
Category Fluency Test	12
CDT	16
CERAD	10
COWAT	7
DRS	7
D-KEFS	5
Digit Span (Forward and Backward)	37
Doors and People Test	2
DSST	18
DTLA	1
EBMT	2
F-A-S Test	4
FCSRT	9
FNAME	2
FRSSD	1
FUCAS	1
GMLT	3
HVLT-R	5
IED	2
Letter Fluency Test	3
List Recall Test	5
List Recognition Test	5
MBT	3
MIST	2
MMSE	120
MoCA	20
NART	5
One Back Test	2
OCL	2
PAL	3
Raven’s Progressive Matrices	2
RVIP	2
RBANS	2
RAVLT	32
ROCF	20
ROCF (Copy)	8
ROCF (Immediate and Delayed Recall)	4
RBMT	3
SRT	7
Semantic Verbal Fluency Test	12
SCWT	23
SDMT	8
TMT-A	60

TMT-B	60
Verbal Fluency Test	23
VLMT	6
Warrington's Recognition Memory Test	2
WAIS-III	5
WAIS-IV	5
WAIS-IV (Block Design Test)	3
WCST-64	4
WMS-III	4
WMS-III (Immediate and Delayed Recall)	2
WMS-III (Logical Memory)	5
WMS-IV	2
WMS-IV (Logical Memory)	3
WMS-R	7
WMS-R (Logical memory)	4
Word List Learn Test (Delayed Recall)	2
Word List Memory	8
Word List Recall Test	7
WST	3
Cognitive tests only used once:	48

Notes: ACE-III = Addenbrooke's Cognitive Examination III, ACE-R = Addenbrooke's Cognitive Examination-Revised, AKT = Alters-Konzentrations-Test, ADAS = Alzheimer's Disease Assessment Scale, Boston Naming Test (BNT), BVRT = Benton Visual Retention Test, CVLT-II = California Verbal Learning Test-Second Edition, CAMCOG = Cambridge Cognitive Examination, CAMCOG-R = Cambridge Cognitive Examination Revised, CCRT = Cambridge Contextual Reading Test, CDT = Clock Drawing Test, CERAD = Consortium to Establish a Registry for Alzheimer's Disease, COWAT = Controlled Oral Word Association Test, DRS = Dementia Rating Scale, D-KEFS = Delis-Kaplan Executive Function System, DSST = Digit Symbol Substitution Test, DTLA = Detection Test for Language impairments in Adults and the Aged, EBMT =East Boston Memory Test, FCSRT = Free and Cued Selective Reminding Test, FNAME = Face-Name Associative Memory Test, FRSSD = Functional Rating Scale for Symptoms of Dementia, FUCAS = Functional Cognitive Assessment Scale, GMLT = Groton Maze Learning Test, HVLT-R = Hopkins Verbal Learning Test-Revised, IED = Intra-Extra Dimensional Set Shift, MBT = Memory Binding Test, MIST = Memory for Intentions Test, MMSE = Mini–Mental State Examination, MoCA = Montreal Cognitive Assessment, NART = National Adult Reading Test, OCL = One Card Learning Test, PAL = Paired Associates Learning, RVIP = Rapid Visual information processing, RBANS = Repeatable Battery for the Assessment of Neuropsychological Status, RAVLT = Rey Auditory Verbal Learning Test, ROCF = Rey-Osterrieth Complex Figure Test, RBMT = Rivermead Behavioural Memory Test, SRT = Selective Reminding Test, SCWT = Stroop Color and Word Test, SDMT = Symbol Digit Modalities Test, TMT-A = Trail Making Test A, TMT-B - Trail Making Test B, VLMT = Verbal Learning and Memory Test, WAIS-III = Wechsler Adult Intelligence Scale Third Edition, WAIS-IV = Wechsler Adult Intelligence Scale Forth Edition, WCST-65 = Wisconsin Card Sorting Test 64 Card Version, WMS-III = Wechsler Memory Scale Third Edition, WMS-IV = Wechsler Memory Scale-Revised Fourth Edition, WMS-R = Wechsler Memory Scale-Revised, WST = Wortschatztest

Cognitive tests only used once include the following = 5-Choice Serial Reaction Time Task (5CSRT), 15 Words Test (15WT), Auditory Consonant Trigrams Test (ACT), Buschke Selective Reminding Test, Camel and Cactus Test (CCT), Choice Reaction Time (CRT), Cognitive Abilities Screening Instrument (CASI), Comprehensive Trail Making Test (CTMT), Corsi Span Task, Delayed-Matching-To-Sample (DMS), Detection Test for Language impairments in Adults and the Aged (DTLA), East Boston Memory Test, Finger-Tapping Test (FTT), Frontal Assessment Battery (FAB), Fuld Object Memory Evaluation (FOME), Functional Rating Scale for Symptoms of Dementia (FRSSD), Functional Cognitive Assessment Scale (FUCAS), General Knowledge Recognition Test, Graded Naming Test (GNT), Hayling and Brixton Tests, Loewenstein-Acevedo Scale for Semantic Interference and Learning (LASSI-L), Lexical Fluency Task, Medical College of Georgia Complex Figures Test, Memory Alteration Test (M@T), Multi-Item Localization task (MILO), Multi-source interference task (MSIT), National Alzheimer's Coordinating Center (NACC) Delayed Paragraph Recall, Neuropsychological Test Battery Vienna (NTBV), One

Touch Stocking of Cambridge (OTS), Pattern Recognition Memory (PRM), Prose Memory Test, Rappel indicé-48, Regensburg Word Fluency Test, Royal Prince Alfred Prospective Memory Test, Short Blessed Test (SBT), Short-Term Memory Binding Task (STMB), Simple Reaction Time (SRT), Spanish and English Neuropsychological Assessment Scales (SENAS), Spatial Recognition Memory (SRM), Spatial Span (SSP), Spatial Working Memory (SWM), Sustained Attention to Response Test (SART), Verbal-Linguistic Intelligence Test, Visual Form Discrimination Test (VFDT), Visual Search Task, Visual Short Term Memory Task, Wechsler Memory Scale-Revised Digit Span (WMS-R Digit Span), Wechsler Test of Adult Reading (WTAR).
