# Supplementary material for: Subjective cognitive decline and objective cognitive performance in older adults: A systematic review of longitudinal and cross‐sectional studies
Source: J Neuropsychol. 2024 Jul 29;19(1):98–114. doi: 10.1111/jnp.12384 (PMC11891377; doi:10.1111/jnp.12384)
Supplement: Supplementary file 6 — Appendix S6. [file JNP-19-98-s004.docx]

**References**

Açikgöz, M., Özen Barut, B., Emre, U., Taşçilar, N., Atalay, A., & Köktürk, F. (2014). Assessment of relation between subjective memory complaints and objective cognitive performance of elderly over 55 years old age. *Noropsikiyatri Arsivi*, *51*(1), 57–62. <https://doi.org/10.4274/npa.y6719>

Amariglio, R. E., Becker, J. A., Carmasin, J., Wadsworth, L. P., Lorius, N., Sullivan, C., Maye, J. E., Gidicsin, C., Pepin, L. C., Sperling, R. A., Johnson, K. A., & Rentz, D. M. (2012). Subjective cognitive complaints and amyloid burden in cognitively normal older individuals. *Neuropsychologia*, *50*(12), 2880–2886. <https://doi.org/10.1016/j.neuropsychologia.2012.08.011>

Amariglio, R. E., Buckley, R. F., Mormino, E. C., Marshall, G. A., Johnson, K. A., Rentz, D. M., & Sperling, R. A. (2018). Amyloid-associated increases in longitudinal report of subjective cognitive complaints. *Alzheimer’s & Dementia: Translational Research & Clinical Interventions*, *4*(1), 444–449. <https://doi.org/10.1016/j.trci.2018.08.005>

Amariglio, R. E., Townsend, M. K., Grodstein, F., Sperling, R. A., & Rentz, D. M. (2011). Specific subjective memory complaints in older persons may indicate poor cognitive function. *Journal of the American Geriatrics Society*, *59*(9), 1612–1617. <https://doi.org/10.1111/j.1532-5415.2011.03543.x>

Aronov, A., Rabin, L. A., Fogel, J., Chi, S. Y., Kann, S. J., Abdelhak, N., & Zimmerman, M. E. (2015). Relationship of cognitive strategy use to prospective memory performance in a diverse sample of nondemented older adults with varying degrees of cognitive complaints and impairment. *Neuropsychology, Development, and Cognition*, *22*(4), 486–501. <https://doi.org/10.1080/13825585.2014.984653>

Bhome, R., Berry, A. J., Huntley, J. D., & Howard, R. J. (2018). Interventions for subjective cognitive decline: Systematic review and meta-analysis. *BMJ Open*, *8*(7). <https://doi.org/10.1136/BMJOPEN-2018-021610>

Boccardi, V., Bubba, V., Murasecco, I., Pigliautile, M., Monastero, R., Cecchetti, R., Scamosci, M., Bastiani, P., Mecocci, P., & Re, G. A. L. study group. (2020). Serum alkaline phosphatase is elevated and inversely correlated with cognitive functions in subjective cognitive decline: Results from the ReGAl 2.0 project. *Aging Clinical and Experimental Research*, *33*, 603-609. <https://doi.org/10.1007/s40520-020-01572-6>

Boggess, M. B., Barber, J. M., Jicha, G. A., & Caban-Holt, A. (2020). Subjective memory complaints are an important surrogate for objective cognitive performance in African Americans. *Alzheimer Disease and Associated Disorders*, *34*(1), 79–84. <https://doi.org/10.1097/WAD.0000000000000348>

Bruce, D. G., Davis, W. A., Hunter, M. L., & Davis, T. M. E. (2019). Subjective memory complaints are not increased in type 2 diabetes: A matched cohort study. *Journal of Diabetes and Its Complications*, *33*(6), 424–426. <https://doi.org/10.1016/j.jdiacomp.2019.04.001>

Brugnolo, A., Morbelli, S., Arnaldi, D., de Carli, F., Accardo, J., Bossert, I., Dessi, B., Fama, F., Ferrara, M., Girtler, N., Picco, A., Rodriguez, G., Sambuceti, G., & Nobili, F. (2014). Metabolic correlates of Rey auditory verbal learning test in elderly subjects with memory complaints. *Journal of Alzheimer’s Disease : JAD*, *39*(1), 103–113. <https://doi.org/10.3233/JAD-121684>

Bruus, A. E., Waldemar, G., & Vogel, A. (2023). Subjective complaints are similar in subjective cognitive decline and early-stage Alzheimer’s disease when assessed in a memory clinic setting. *Journal of Geriatric Psychiatry and Neurology*, *0*(0). <https://doi.org/10.1177/08919887231164352>

Buckley, R. F., Maruff, P., Ames, D., Bourgeat, P., Martins, R. N., Masters, C. L., Rainey-Smith, S., Lautenschlager, N., Rowe, C. C., Savage, G., Villemagne, V. L., Ellis, K. A., & study, A. (2016). Subjective memory decline predicts greater rates of clinical progression in preclinical Alzheimer’s disease. *Alzheimer’s & Dementia: The Journal of the Alzheimer’s Association*, *12*(7), 796–804. <https://doi.org/10.1016/j.jalz.2015.12.013>

Buckley, R. F., Saling, M. M., Irish, M., Ames, D., Rowe, C. C., Lautenschlager, N. T., Maruff, P., Macaulay, S. L., Martins, R. N., Masters, C. L., Rainey-Smith, S. R., Rembach, A., Savage, G., Szoeke, C., & Ellis, K. A. (2014). Personal memory function in mild cognitive impairment and subjective memory complaints: Results from the Australian Imaging, Biomarkers, and Lifestyle (AIBL) Study of Ageing. *Journal of Alzheimer’s Disease: JAD*, *40*(3), 551–561. <https://doi.org/10.3233/JAD-131820>

Burmester, B., Leathem, J., & Merrick, P. (2016). Subjective cognitive complaints and objective cognitive function in aging: A systematic review and meta-analysis of recent cross-sectional findings. *Neuropsychology Review*, *26*(4), 376–393. <https://doi.org/10.1007/s11065-016-9332-2>

Caillaud, M., Hudon, C., Boller, B., Brambati, S., Duchesne, S., Lorrain, D., Gagnon, J.-F., Maltezos, S., Mellah, S., Phillips, N., Consortium for the Early Identification of Alzheimer’s Disease-Quebec, & Belleville, S. (2020). Evidence of a relation between hippocampal volume, white matter hyperintensities, and cognition in subjective cognitive decline and mild cognitive impairment. *The Journals of Gerontology. Series B, Psychological Sciences and Social Sciences*, *75*(7), 1382–1392.<https://doi.org/10.1093/geronb/gbz120>

Cantero, J. L., Iglesias, J. E., van Leemput, K., & Atienza, M. (2016). Regional hippocampal atrophy and higher levels of plasma amyloid-beta are associated with subjective memory complaints in nondemented elderly subjects. *The Journals of Gerontology*, *71*(9), 1210–1215. <https://doi.org/10.1093/gerona/glw022>

Carmasin, J. S., Roth, R. M., Rabin, L. A., Englert, J. J., Flashman, L. A., & Saykin, A. J. (2021). Stability of subjective executive functioning in older adults with aMCI and subjective cognitive decline. *Archives of Clinical Neuropsychology : The Official Journal of the National Academy of Neuropsychologists*, *36*(6), 1012–1018.<https://doi.org/10.1093/arclin/acaa129>

Carp, F. M. (1989). Maximizing data quality in community studies of older people. In: M.P. Lawton & A.R. Herzog (Eds.), *Special Research Methods for Gerontology* (pp. 93-122). Baywood Publishing Co. Inc.

Carr, S., Pichora-Fuller, M. K., Li, K. Z. H., Phillips, N., & Campos, J. L. (2019). Multisensory, multi-tasking performance of older adults with and without subjective cognitive decline. *Multisensory Research*, 32(8), 797-829. <https://doi.org/10.1163/22134808-20191426>

Cerman, J., Andel, R., Laczo, J., Vyhnalek, M., Nedelska, Z., Mokrisova, I., Sheardova, K., & Hort, J. (2018). Subjective spatial navigation complaints - A frequent symptom reported by patients with subjective cognitive decline, mild cognitive impairment and Alzheimer’s disease. *Current Alzheimer Research*, *15*(3), 219–228. <https://doi.org/10.2174/1567205014666171120145349>

Chamberlain, S. R., Blackwell, A. D., Nathan, P. J., Hammond, G., Robbins, T. W., Hodges, J. R., Michael, A., & Semple, J. M. (2015). Differential cognitive deterioration in dementia: A two year longitudinal study. *Advances in Alzheimer’s Disease*, *4*, 107–118. <https://doi.org/10.3233/978-1-61499-542-5-107>

Chang, H.-T., Chen, T.-F., Cheng, T.-W., Lai, Y.-M., & Hua, M.-S. (2018). Arbitrary and semantic associations in subjective memory impairment and amnestic mild cognitive impairment among Taiwanese individuals: A cross-sectional study. *Journal of the Formosan Medical Association*, *117*(5), 427–433. <https://doi.org/10.1016/j.jfma.2017.05.014>

Chang, K. H., Wang, C., Nester, C. O., Katz, M. J., Byrd, D. A., Lipton, R. B., & Rabin, L. A. (2023). Examining the role of participant and study partner report in widely-used classification approaches of mild cognitive impairment in demographically-diverse community dwelling individuals: results from the Einstein aging study. *Frontiers in Aging Neuroscience*, *15*, 1221768. https://doi.org/10.3389/fnagi.2023.1221768

Chapman, S., Sunderaraman, P., Joyce, J. L., Azar, M., Colvin, L. E., Barker, M. S., McKeague, I., Kreisl, W. C., & Cosentino, S. (2021). Optimizing subjective cognitive decline to detect early cognitive dysfunction. *Journal of Alzheimer’s Disease : JAD*, *80*(3), 1185–1196.<https://doi.org/10.3233/JAD-201322>

Chen Q., Lu J., Zhang X., Sun Y., Chen W., Li X., … Zhang B. (2021). Alterations in dynamic functional connectivity in individuals with subjective cognitive decline. *Frontiers in Aging Neuroscience*, *13*, 646017.<https://doi.org/10.3389/fnagi.2021.646017>

Chen, Q., Wu, S., Li, X., Sun, Y., Chen, W., Lu, J., ... Zhang, B. (2021). Basal forebrain atrophy is associated with allocentric navigation deficits in subjective cognitive decline. *Frontiers in Aging Neuroscience, 13*, 596025. <https://doi.org/10.3389/fnagi.2021.596025>

Cheng, G., Liu, D., Huang, L., Han, G., Hu, F., Wu, Z., … Zeng, Y. (2023). Prevalence and risk factors for subjective cognitive decline and the correlation with objective cognition among community-dwelling older adults in china: Results from the hubei memory and aging cohort study. *Alzheimer’s & Dementia: The Journal of the Alzheimer’s Association*, <https://doi.org/10.1002/alz.13047>

Chi, S. Y., Chua, E. F., Kieschnick, D. W., & Rabin, L. A. (2020). Retrospective metamemory monitoring of semantic memory in community-dwelling older adults with subjective cognitive decline and mild cognitive impairment. *Neuropsychological Rehabilitation*, *32*(3), 429-463. <https://doi.org/10.1080/09602011.2020.1831552>

Chi, S. Y., Rabin, L. A., Aronov, A., Fogel, J., Kapoor, A., & Wang, C. (2014). Differential focal and nonfocal prospective memory accuracy in a demographically diverse group of nondemented community-dwelling older adults. *Journal of the International Neuropsychological Society: JINS*, *20*(10), 1015–1027. <https://doi.org/10.1017/S1355617714000964>

Chin, J., Oh, K. J., Seo, S. W., & Na, D. L. (2014). Are depressive symptomatology and self-focused attention associated with subjective memory impairment in older adults? *International Psychogeriatrics*, *26*(4), 573–580. <https://doi.org/10.1017/S104161021300241X>

Choe, Y. M., Byun, M. S., Lee, J. H., Sohn, B. K., Lee, D. Y., & Kim, J. W. (2018). Subjective memory complaint as a useful tool for the early detection of Alzheimer’s disease. *Neuropsychiatric Disease and Treatment*, *14*, 2451–2460. <https://doi.org/10.2147/NDT.S174517>

Corlier, F. W., Shaw, C., Hayes-Larson, E., Mungas, D., Tomaszewski Farias, S., Glymour, M. M., Whitmer, R. A., & Mayeda, E. R. (2020). Association between cognitive test performance and subjective cognitive decline in a diverse cohort of older adults: Findings from the KHANDLE study. *Alzheimer Disease and Associated Disorders*, *34*(3), 198–205. <https://doi.org/10.1097/WAD.0000000000000383>

Crocco, E. A., Loewenstein, D. A., Curiel, R. E., Alperin, N., Czaja, S. J., Harvey, P. D., Sun, X. Y., Lenchus, J., Raffo, A., Penate, A., Melo, J., Sang, L., Valdivia, R., & Cardenas, K. (2018). A novel cognitive assessment paradigm to detect Pre-mild cognitive impairment (PreMCI) and the relationship to biological markers of Alzheimer’s disease. *Journal of Psychiatric Research*, *96*, 33–38. <https://doi.org/10.1016/j.jpsychires.2017.08.015>

Crumley, J. J., Stetler, C. A., & Horhota, M. (2014). Examining the relationship between subjective and objective memory performance in older adults: A meta-analysis. *Psychology and Aging*, *29*(2), 250–263. <https://doi.org/10.1037/a0035908>

Cuetos, F., Rodriguez-Ferreiro, J., & Menendez, M. (2009). Semantic markers in the diagnosis of neurodegenerative dementias. *Dementia and Geriatric Cognitive Disorders*, *28*(3), 267–274. <https://doi.org/10.1159/000242438>

Dalpubel, D., Rossi, P. G., de Almeida, M. L., Ribeiro, E. B., Araujo, R., de Andrade, L. P., & do Vale, F. de A. C. (2019). Subjective memory complaint and its relationship with cognitive changes and physical vulnerability of community-dwelling older adults. *Dementia & Neuropsychologia*, *13*(3), 343–349. <https://doi.org/10.1590/1980-57642018dn13-030012>

de Almeida, M. L., Dalpubel, D., Ribeiro, E. B., de Oliveira, E. S. B., Ansai, J. H., & Vale, F. A. C. (2019). Subjective cognitive impairment, cognitive disorders and self-perceived health: The importance of the informant. *Dementia & Neuropsychologia*, *13*(3), 335–342. <https://doi.org/10.1590/1980-57642018dn13-030011>

Dhilla Albers, A., Asafu-Adjei, J., Delaney, M. K., Kelly, K. E., Gomez-Isla, T., Blacker, D., Johnson, K. A., Sperling, R. A., Hyman, B. T., Betensky, R. A., Hastings, L., & Albers, M. W. (2016). Episodic memory of odors stratifies Alzheimer biomarkers in normal elderly. *Annals of Neurology*, *80*(6), 846–857. <https://doi.org/10.1002/ana.24792>

Donovan, N. J., Amariglio, R. E., Zoller, A. S., Rudel, R. K., Gomez-Isla, T., Blacker, D., Hyman, B. T., Locascio, J. J., Johnson, K. A., Sperling, R. A., Marshall, G. A., & Rentz, D. M. (2014). Subjective cognitive concerns and neuropsychiatric predictors of progression to the early clinical stages of Alzheimer disease. *The American Journal of Geriatric Psychiatry: Official Journal of the American Association for Geriatric Psychiatry*, *22*(12), 1642–1651. <https://doi.org/10.1016/j.jagp.2014.02.007>

Dufouil, C., Fuhrer, R., & Alperovitch, A. (2005). Subjective cognitive complaints and cognitive decline: consequence or predictor? The epidemiology of vascular aging study. *Journal of the American Geriatrics Society*, *53*(4), 616–621. <https://doi.org/10.1111/j.1532-5415.2005.53209.x>

Dux, M. C., Woodard, J. L., Calamari, J. E., Messina, M., Arora, S., Chik, H., & Pontarelli, N. (2008). The moderating role of negative affect on objective verbal memory performance and subjective memory complaints in healthy older adults. *Journal of the International Neuropsychological Society: JINS*, *14*(2), 327–336. <https://doi.org/10.1017/S1355617708080363>

Erk, S., Spottke, A., Meisen, A., Wagner, M., Walter, H., & Jessen, F. (2011). Evidence of neuronal compensation during episodic memory in subjective memory impairment. *Archives of General Psychiatry*, *68*(8), 845–852. <https://doi.org/10.1001/archgenpsychiatry.2011.80>

Gardener, S. L., Sohrabi, H. R., Shen, K.-K., Rainey-Smith, S. R., Weinborn, M., Bates, K. A., Shah, T., Foster, J. K., Lenzo, N., Salvado, O., Laske, C., Laws, S. M., Taddei, K., Verdile, G., & Martins, R. N. (2016). Cerebral glucose metabolism is associated with verbal but not visual memory performance in community-dwelling older adults. *Journal of Alzheimer’s Disease: JAD*, *52*(2), 661–672. <https://doi.org/10.3233/JAD-151084>

Gifford K.A., Liu D., Carmona H., Lu Z., Romano R., Tripodis Y., Martin B., Kowall N., & Jefferson A.L. (2014). Inclusion of an informant yields strong associations between cognitive complaint and longitudinal cognitive outcomes in non-demented elders. *Journal of Alzheimer’s Disease*, *43*(1), 121–132.<https://doi.org/10.3233/JAD-131925>

Goda, A., Murata, S., Nakano, H., Nonaka, K., Iwase, H., Shiraiwa, K., Abiko, T., Anami, K., & Horie, J. (2020). The relationship between subjective cognitive decline and health literacy in healthy community-dwelling older adults. *Healthcare (Basel, Switzerland)*, *8*(4).<https://doi.org/10.3390/healthcare8040567>

Goda, A., Murata, S., Nakano, H., Shiraiwa, K., Abiko, T., Nonaka, K., Iwase, H., Anami, K., & Horie, J. (2020). Subjective and objective mental and physical functions affect subjective cognitive decline in community-dwelling elderly Japanese people. *Healthcare (Basel, Switzerland)*, *8*(3).<https://doi.org/10.3390/healthcare8030347>

Hackett, K., Krikorian, R., Giovannetti, T., Melendez-Cabrero, J., Rahman, A., Caesar, E. E., Chen, J. L., Hristov, H., Seifan, A., Mosconi, L., & Isaacson, R. S. (2018). Utility of the NIH Toolbox for assessment of prodromal Alzheimer’s disease and dementia. *Alzheimer’s and Dementia: Diagnosis, Assessment and Disease Monitoring*, *10*, 764–772. <https://doi.org/10.1016/j.dadm.2018.10.002>

Hall, J. R., Wiechmann, A., Johnson, L. A., Edwards, M., & O’Bryant, S. E. (2018). Characteristics of cognitively normal Mexican-Americans with cognitive complaints. *Journal of Alzheimer’s Disease: JAD*, *61*(4), 1485–1492. <https://doi.org/10.3233/JAD-170836>

Hanninen, T., Reinikainen, K. J., Helkala, E. L., Koivisto, K., Mykkanen, L., Laakso, M., Pyorala, K., & Riekkinen, P. J. (1994). Subjective memory complaints and personality traits in normal elderly subjects. *Journal of the American Geriatrics Society*, *42*(1), 1–4. <https://doi.org/10.1111/j.1532-5415.1994.tb06064.x>

Hao, L., Sun, Y., Li, Y., Wang, J., Wang, Z., Zhang, Z., Wei, Z., Gao, G., Jia, J., Xing, Y., & Han, Y. (2020). Demographic characteristics and neuropsychological assessments of subjective cognitive decline (SCD) (plus). *Annals of Clinical and Translational Neurology*, *7*(6), 1002–1012. <https://doi.org/10.1002/acn3.51068>

Hayes, J. M., Tang, L., Viviano, R. P., van Rooden, S., Ofen, N., & Damoiseaux, J. S. (2017). Subjective memory complaints are associated with brain activation supporting successful memory encoding. *Neurobiology of Aging*, *60*, 71–80. <https://doi.org/10.1016/j.neurobiolaging.2017.08.015>

Hays, C. C., Zlatar, Z. Z., Campbell, L., Meloy, M. J., & Wierenga, C. E. (2018). Subjective cognitive decline modifies the relationship between cerebral blood flow and memory function in cognitively normal older adults. *Journal of the International Neuropsychological Society: JINS*, *24*(3), 213–223. <https://doi.org/10.1017/S135561771700087X>

Hill, N. L., Mogle, J., Wion, R., Munoz, E., DePasquale, N., Yevchak, A. M., & Parisi, J. M. (2016). Subjective cognitive impairment and affective symptoms: A systematic review. *Gerontologist*, *56*(6), e109–e127. <https://doi.org/10.1093/geront/gnw091>

Hong, Y. J., Park, J. W., Lee, S. B., Kim, S. H., Kim, Y., Ryu, D. W., ... Yang, D. W. (2021). The influence of amyloid burden on cognitive decline over 2 years in older adults with subjective cognitive decline: A prospective cohort study. *Dementia and Geriatric Cognitive Disorders*, *50*(5), 437-445. <https://doi.org/10.1159/000519766>

Hoops, S., Nazem, S., Siderowf, A., Duda, J., Xie, S., Stern, M., & Weintraub, D. (2009). Validity of the MoCA and MMSE in the detection of MCI and dementia in Parkinson disease. *Neurology*, *73*(21), 1738–1745. <https://doi.org/10.1212/WNL.0b013e3181c34b47>

Hwang, J., Jeong, J. H., Yoon, S. J., Park, K. W., Kim, E. J., Yoon, B., Jang, J. W., Kim, H. J., Hong, J. Y., Lee, J. M., Park, H., Kang, J. H., Choi, Y. H., Park, G., Hong, J., Byun, M. S., Yi, D., Kim, Y. K., Lee, D. Y., & Choi, S. H. (2019). Clinical and biomarker characteristics according to clinical spectrum of Alzheimer’s disease (AD) in the validation cohort of Korean Brain Aging study for the early diagnosis and prediction of AD. *Journal of Clinical Medicine*, *8*(3), 341. <https://doi.org/10.3390/jcm8030341>

Ivanoiu, A., Adam, S., der Linden, M., Salmon, E., Juillerat, A.-C., Mulligan, R., & Seron, X. (2005). Memory evaluation with a new cued recall test in patients with mild cognitive impairment and Alzheimer’s disease. *Journal of Neurology*, *252*(1), 47–55. <https://doi.org/10.1007/s00415-005-0597-2>

Jenkins, A., Tree, J. J., Thornton, I. M., & Tales, A. (2019). Subjective cognitive impairment in 55-65-year-old adults is associated with negative affective symptoms, neuroticism, and poor quality of life. *Journal of Alzheimer’s Disease: JAD*, *67*(4), 1367–1378. <https://doi.org/10.3233/JAD-180810>

Jeong, H. S., Park, J. S., Song, I. U., Chung, Y. A., & Rhie, S. J. (2017). Changes in cognitive function and brain glucose metabolism in elderly women with subjective memory impairment: A 24-month prospective pilot study. *Acta Neurologica Scandinavica*, *135*(1), 108–114. <https://doi.org/10.1111/ane.12569>

Jessen, F. (2014). Subjective and objective cognitive decline at the pre-dementia stage of Alzheimer’s disease. *European Archives of Psychiatry and Clinical Neuroscience*, *264*(1), 3–7. <https://doi.org/10.1007/s00406-014-0539-z>

Jessen, F., Amariglio, R. E., Boxtel, M. van, Breteler, M., Ceccaldi, M., Chételat, G., Dubois, B., Dufouil, C., Ellis, K. A., Flier, W. M. van der, Glodzik, L., Harten, A. C. van, Leon, M. J. de, McHugh, P., Mielke, M. M., Molinuevo, J. L., Mosconi, L., Osorio, R. S., Perrotin, A., … Group, S. C. D. I. (SCD-I. W. (2014). A conceptual framework for research on subjective cognitive decline in preclinical Alzheimer’s disease. *Alzheimer’s & Dementia: The Journal of the Alzheimer’s Association*, *10*(6), 844. <https://doi.org/10.1016/J.JALZ.2014.01.001>

Jessen, F., Amariglio, R. E., Buckley, R. F., van der Flier, W. M., Han, Y., Molinuevo, J. L., Rabin, L., Rentz, D. M., Rodriguez-Gomez, O., Saykin, A. J., Sikkes, S. A. M., Smart, C. M., Wolfsgruber, S., & Wagner, M. (2020). The characterisation of subjective cognitive decline. *The Lancet Neurology*, *19*(3), 271–278. <https://doi.org/10.1016/S1474-4422(19)30368-0>

Jessen, F., Spottke, A., Boecker, H., Brosseron, F., Buerger, K., Catak, C., Fliessbach, K., Franke, C., Fuentes, M., Heneka, M. T., Janowitz, D., Kilimann, I., Laske, C., Menne, F., Nestor, P., Peters, O., Priller, J., Pross, V., Ramirez, A., … Duzel, E. (2018). Design and first baseline data of the DZNE multicenter observational study on predementia Alzheimer’s disease (DELCODE). *Alzheimer’s Research & Therapy*, *10*(1), 15. <https://doi.org/10.1186/s13195-017-0314-2>

Jonker, C., Geerlings, M. I., & Schmand, B. (2000). Are memory complaints predictive for dementia: A review of clinical and population-based studies. *International Journal of Geriatric* Psychiatry, *872*(880), 983–991. [https://doi.org/10.1002/1099-1166(200011)15:11<983::aid-gps238>3.0.co;2-5](https://doi.org/10.1002/1099-1166(200011)15:11%3c983::aid-gps238%3e3.0.co;2-5)

Jonker, C., Launer, L. J., Hooijer, C., & Lindeboom, J. (1996). Memory complaints and memory impairment in older individuals. *Journal of the American Geriatrics Society*, *44*(1), 44–49. <https://doi.org/10.1111/j.1532-5415.1996.tb05636.x>

Jungwirth, S., Fischer, P., Weissgram, S., Kirchmeyr, W., Bauer, P., & Tragl, K.-H. (2004). Subjective memory complaints and objective memory impairment in the Vienna-Transdanube aging community. *Journal of the American Geriatrics Society*, *52*(2), 263–268. <https://doi.org/10.1111/j.1532-5415.2004.52066.x>

Kamberis, N., Cavuoto, M. G., & Pike, K. E. (2021). The influence of subjective cognitive decline on prospective memory over 5 years. *Neuropsychology*, *35*(1), 78–89.<https://doi.org/10.1037/neu0000709>

Katayama, O., Lee, S., Bae, S., Makino, K., Chiba, I., Harada, K., Morikawa, M., Tomida, K., & Shimada, H. (2022). Differences in subjective and objective cognitive decline outcomes are associated with modifiable protective factors: A 4-year longitudinal study. *Journal of Clinical Medicine*, *11*(24). <https://doi.org/10.3390/jcm11247441>

Kielb, S., Rogalski, E., Weintraub, S., & Rademaker, A. (2017). Objective features of subjective cognitive decline in a United States national database. *Alzheimer’s & Dementia: The Journal of the Alzheimer’s Association*, *13*(12), 1337–1344. <https://doi.org/10.1016/j.jalz.2017.04.008>

Kim, D., Lee, S., Choi, M., Youn, H., Suh, S., Jeong, H.-G., & Han, C. E. (2019). Diffusion tensor imaging reveals abnormal brain networks in elderly subjects with subjective cognitive deficits. *Neurological Sciences*, *40*(11), 2333–2342. <https://doi.org/10.1007/s10072-019-03981-6>

Kim, W.-H., Kim, B.-S., Chang, S.-M., Lee, D.-W., & Bae, J.-N. (2020). Relationship between subjective memory complaint and executive function in a community sample of South Korean elderly. *Psychogeriatrics : The Official Journal of the Japanese Psychogeriatric Society*, *20*(6), 850–857.<https://doi.org/10.1111/psyg.12592>

Kirsebom, B. E., Nordengen, K., Selnes, P., Waterloo, K., Torsetnes, S. B., Gisladottir, B., Brix, B., Vanmechelen, E., Brathen, G., Hessen, E., Aarsland, D., & Fladby, T. (2018). Cerebrospinal fluid neurogranin/beta-site APP-cleaving enzyme 1 predicts cognitive decline in preclinical Alzheimer’s disease. *Alzheimer’s and Dementia: Translational Research and Clinical Interventions*, *4*, 617–627. <https://doi.org/10.1016/j.trci.2018.10.003>

Koppara, A., Frommann, I., Polcher, A., Parra, M. A., Maier, W., Jessen, F., Klockgether, T., & Wagner, M. (2015). Feature binding deficits in subjective cognitive decline and in mild cognitive impairment. *Journal of Alzheimer’s Disease*, *48*(S1), S161–S170. <https://doi.org/10.3233/JAD-150105>

Koppara, A., Wagner, M., Lange, C., Ernst, A., Wiese, B., Konig, H.-H., Brettschneider, C., Riedel-Heller, S., Luppa, M., Weyerer, S., Werle, J., Bickel, H., Mosch, E., Pentzek, M., Fuchs, A., Wolfsgruber, S., Beauducel, A., Scherer, M., Maier, W., & Jessen, F. (2015). Cognitive performance before and after the onset of subjective cognitive decline in old age. *Alzheimer’s & Dementia*, *1*(2), 194–205. <https://doi.org/10.1016/j.dadm.2015.02.005>

Koyama, A., Steinman, M., Ensrud, K., Hillier, T. A., & Yaffe, K. (2014). Long-term cognitive and functional effects of potentially inappropriate medications in older women. *The Journals of Gerontology. Series A, Biological Sciences and Medical Sciences, 69*(4), 423–429. https://doi.org/10.1093/gerona/glt192

Kramberger, M. G., Jelic, V., Kareholt, I., Enache, D., Eriksdotter Jonhagen, M., Winblad, B., & Aarsland, D. (2012). Cerebrospinal fluid Alzheimer markers in depressed elderly subjects with and without Alzheimer’s disease. *Dementia and Geriatric Cognitive Disorders Extra*, *2*(1), 48–56. <https://doi.org/10.1159/000334644>

La Joie, R., Perrotin, A., Egret, S., Pasquier, F., Tomadesso, C., Mézenge, F., Desgranges, B., de La Sayette, V., & Chételat, G. (2016). Qualitative and quantitative assessment of self-reported cognitive difficulties in nondemented elders: Association with medical help seeking, cognitive deficits, and β-amyloid imaging. *Alzheimer’s & Dementia: Diagnosis, Assessment & Disease Monitoring*, *5*, 23–34. <https://doi.org/10.1016/j.dadm.2016.12.005>

Laws, S. M., Clarnette, R. M., Taddei, K., Martins, G., Paton, A., Hallmayer, J., Almeida, O. P., Groth, D. M., Gandy, S. E., Forstl, H., & Martins, R. N. (2002). APOE-epsilon 4 and APOE-491A polymorphisms in individuals with subjective memory loss. *Molecular Psychiatry*, *7*(7), 768–775. <https://doi.org/10.1038/sj.mp.4001083>

Lazarou, I., Moraitou, D., Papatheodorou, M., Vavouras, I., Lokantidou, C., Agogiatou, C., Gialaoutzis, M., Nikolopoulos, S., Stavropoulos, T. G., Kompatsiaris, I., & Tsolaki, M. (2021). Adaptation and validation of the memory alteration test (M@T) in Greek middle-aged, older, and older-old population with subjective cognitive decline and mild cognitive impairment. *Journal of Alzheimer’s Disease : JAD*, *84*(3), 1219–1232.<https://doi.org/10.3233/JAD-210558>

Lazarou I., Georgiadis K., Nikolopoulos S., Oikonomou V.P., Stavropoulos T.G., Tsolaki A., Kompatsiaris I., & Tsolaki M. (2022). Exploring network properties across preclinical stages of Alzheimer’s disease using a visual short-term memory and attention task with high-density electroencephalography: A brain-connectome neurophysiological study. *Journal of Alzheimer’s Disease*, *87*(2), 643–664. <https://doi.org/10.3233/JAD-215421>

Lee, S. D., Ong, B., Pike, K. E., & Kinsella, G. J. (2018). Prospective memory and subjective memory decline: A neuropsychological indicator of memory difficulties in community-dwelling older people. *Journal of Clinical and Experimental Neuropsychology*, *40*(2), 183–197. <https://doi.org/10.1080/13803395.2017.1326465>

Lehrner, J., Bodendorfer, B., Lamm, C., Moser, D., Dal-Bianco, P., Auff, E., & Pusswald, G. (2016). Subjective memory complaints and conversion to dementia in patients with subjective cognitive decline and patients with mild cognitive impairment. *Zeitschrift Fur Neuropsychologie*, *27*(2), 85–93. <https://doi.org/10.1024/1016-264X/a000175>

Lehrner, J., Coutinho, G., Mattos, P., Moser, D., Pfluger, M., Gleiss, A., Auff, E., Dal-Bianco, P., Pusswald, G., & Stogmann, E. (2017). Semantic memory and depressive symptoms in patients with subjective cognitive decline, mild cognitive impairment, and Alzheimer’s disease. *International Psychogeriatrics*, *29*(7), 1123–1135. <https://doi.org/10.1017/S1041610217000394>

Lehrner, J., Kogler, S., Lamm, C., Moser, D., Klug, S., Pusswald, G., Dal-Bianco, P., Pirker, W., & Auff, E. (2015). Awareness of memory deficits in subjective cognitive decline, mild cognitive impairment, Alzheimer’s disease and Parkinson’s disease. *International Psychogeriatrics*, *27*(3), 357–366. <https://doi.org/10.1017/S1041610214002245>

Li, W., Yue, L., & Xiao, S. (2022). Subjective cognitive decline is associated with a higher risk of objective cognitive decline: A cross-sectional and longitudinal study. *Frontiers in Psychiatry*, *13*(101545006), 950270. <https://doi.org/10.3389/fpsyt.2022.950270>

Liang L., Yuan Y., Wei Y., Yu B., Mai W., Duan G., Nong X., Li C., Su J., Zhao L., Zhang Z., & Deng D. (2021). Recurrent and concurrent patterns of regional BOLD dynamics and functional connectivity dynamics in cognitive decline. *Alzheimer’s Research and Therapy*, *13*(1), 28.<https://doi.org/10.1186/s13195-020-00764-6>

Liew, T. M., Yap, P., Ng, T. P., Mahendran, R., Kua, E. H., & Feng, L. (2019). Symptom clusters of subjective cognitive decline amongst cognitively normal older persons and their utilities in predicting objective cognitive performance: Structural equation modelling. *European Journal of Neurology*, *26*(9), 1153–1160. <https://doi.org/10.1111/ene.13958>

Liu, Y., Li, Z., Jiang, X., Du, W., Wang, X., Sheng, C., Jiang, J., & Han, Y. (2021). Differences in functional brain networks between subjective cognitive decline with and without worry groups: A graph theory study from SILCODE. *Journal of Alzheimer’s Disease : JAD*, *84*(3), 1279–1289.<https://doi.org/10.3233/JAD-215156>

Lubitz, A. F., Eid, M., & Niedeggen, M. (2020). Psychosocial and cognitive performance correlates of subjective cognitive complaints in help-seeking versus non-help-seeking community-dwelling adults. *Journal of Geriatric Psychiatry and Neurology*, *33*(2), 93–102. <https://doi.org/10.1177/0891988719856691>

Macoir, J., Lafay, A., & Hudon, C. (2019). Reduced lexical access to verbs in individuals with subjective cognitive decline. *American Journal of Alzheimer’s Disease and Other Dementias*, *34*(1), 5–15. <https://doi.org/10.1177/1533317518790541>

Macoir, J., Tremblay, P., & Hudon, C. (2022). The use of executive fluency tasks to detect cognitive impairment in individuals with subjective cognitive decline. *Behavioral Sciences*, *12*(12). <https://doi.org/10.3390/bs12120491>

Maguire, F. J., Killane, I., Creagh, A. P., Donoghue, O., Kenny, R. A., & Reilly, R. B. (2018). Baseline association of motoric cognitive risk syndrome with sustained attention, memory, and global cognition. *Journal of the American Medical Directors Association*, *19*(1), 53–58. <https://doi.org/10.1016/j.jamda.2017.07.016>

Mallo, S. C., Ismail, Z., Pereiro, A. X., Facal, D., Lojo-Seoane, C., Campos-Magdaleno, M., & Juncos-Rabadan, O. (2019). Assessing mild behavioral impairment with the mild behavioral impairment checklist in people with subjective cognitive decline. *International Psychogeriatrics*, *31*(2), 231–239. <https://doi.org/10.1017/S1041610218000698>

Manousakis, J. E., Scovelle, A. J., Rajaratnam, S. M. W., Naismith, S. L., & Anderson, C. (2018). Advanced circadian timing and sleep fragmentation differentially impact on memory complaint subtype in subjective cognitive decline. *Journal of Alzheimer’s Disease: JAD*, *66*(2), 565–577. <https://doi.org/10.3233/JAD-180612>

Markova, H., Andel, R., Stepankova, H., Kopecek, M., Nikolai, T., Hort, J., Thomas-Anterion, C., & Vyhnalek, M. (2017). Subjective cognitive complaints in cognitively healthy older adults and their relationship to cognitive performance and depressive symptoms. *Journal of Alzheimer’s Disease: JAD*, *59*(3), 871–881. <https://doi.org/10.3233/JAD-160970>

Markova, H., Fendrych Mazancova, A., Jester, D. J., Cechova, K., Matuskova, V., Nikolai, T., Nedelska, Z., Uller, M., Andel, R., Laczo, J., Hort, J., & Vyhnalek, M. (2022). Memory binding test and its associations with hippocampal volume across the cognitive continuum preceding dementia. *Assessment*, *30*(3), 856–872.<https://doi.org/10.1177/10731911211069676>

Markova, H., Nikolai, T., Mazancova, A. F., Cechova, K., Sheardova, K., Georgi, H., Kopecek, M., Laczo, J., Hort, J., & Vyhnalek, M. (2019). Differences in subjective cognitive complaints between non-demented older adults from a memory clinic and the community. *Journal of Alzheimer’s Disease: JAD*, *70*(1), 61–73. <https://doi.org/10.3233/JAD-180630>

Merema, M. R., Speelman, C. P., Foster, J. K., & Kaczmarek, E. A. (2013). Neuroticism (not depressive symptoms) predicts memory complaints in some community-dwelling older adults. *The American Journal of Geriatric Psychiatry: Official Journal of the American Association for Geriatric Psychiatry*, *21*(8), 729–736. <https://doi.org/10.1016/j.jagp.2013.01.059>

Minett, T. S. C., da Silva, R. V., Ortiz, K. Z., & Bertolucci, P. H. F. (2008). Subjective memory complaints in an elderly sample: A cross-sectional study. *International Journal of Geriatric Psychiatry*, *23*(1), 49–54. <https://doi.org/10.1002/gps.1836>

Mogre, V., Johnson, N. A., Tzelepis, F., Shaw, J. E., & Paul, C. (2019). A systematic review of adherence to diabetes self-care behaviours: Evidence from low- and middle-income countries. *Journal of Advanced Nursing*, *75*(12), 3374–3389. <https://doi.org/10.1111/JAN.14190>

Mol, M. E. M., van Boxtel, M. P. J., Willems, D., & Jolles, J. (2006). Do subjective memory complaints predict cognitive dysfunction over time? A six-year follow-up of the Maastricht Aging Study. *International Journal of Geriatric Psychiatry*, *21*(5), 432–441. <https://doi.org/10.1002/gps.1487>

Molinuevo, J. L., Rabin, L. A., Amariglio, R., Buckley, R., Dubois, B., Ellis, K. A., Ewers, M., Hampel, H., Klöppel, S., Rami, L., Reisberg, B., Saykin, A. J., Sikkes, S., Smart, C. M., Snitz, B. E., Sperling, R., van der Flier, W. M., Wagner, M., & Jessen, F. (2017). Implementation of subjective cognitive decline criteria in research studies. *Alzheimer’s and Dementia*, *13*(3), 296–311. <https://doi.org/10.1016/j.jalz.2016.09.012>

Montejo Carrasco, P., Montenegro-Pena, M., Lopez-Higes, R., Estrada, E., Prada Crespo, D., Montejo Rubio, C., & Garcia Azorin, D. (2017). Subjective memory complaints in healthy older adults: Fewer complaints associated with depression and perceived health, more complaints also associated with lower memory performance. *Archives of Gerontology and Geriatrics*, *70*, 28–37. <https://doi.org/10.1016/j.archger.2016.12.007>

Montejo, P., Montenegro, M., Fernandez-Blazquez, M. A., Turrero-Nogues, A., Yubero, R., Huertas, E., & Maestu, F. (2014). Association of perceived health and depression with older adults’ subjective memory complaints: contrasting a specific questionnaire with general complaints questions. *European Journal of Ageing*, *11*(1), 77–87. <https://doi.org/10.1007/s10433-013-0286-4>

Morrison, C., Dadar, M., Shafiee, N., Villeneuve, S., & Collins, D. L. (2022). Regional brain atrophy and cognitive decline depend on definition of subjective cognitive decline. *NeuroImage Clinical*, *33*, 102923.<https://doi.org/10.1016/j.nicl.2021.102923>

Morrison, C., Dadar, M., Shafiee, N., & Collins, D. L. (2023). The use of hippocampal grading as a biomarker for preclinical and prodromal Alzheimer's disease. *Human Brain Mapping*, 44(8), 3147– 3157. <https://doi.org/10.1002/hbm.26269>

Morrison, C. & Oliver, M. D. (2023). Subjective cognitive decline is associated with lower baseline cognition and increased rate of cognitive decline. *The Journals of Gerontology. Series B, Psychological Sciences and Social Sciences*, *78*(4), 573–584. <https://doi.org/10.1093/geronb/gbac178>

Moulinet, I., Touron, E., Mézenge, F., Dautricourt, S., De La Sayette, V., Vivien, D., Marchant, N. L., Poisnel, G., & Chételat, G. (2022). Depressive symptoms have distinct relationships with neuroimaging biomarkers across the Alzheimer’s clinical continuum. *Frontiers in Aging Neuroscience*, 1–12.<https://doi.org/10.3389/fnagi.2022.899158>

Mulligan, B. P., Smart, C. M., & Ali, J. I. (2016). Relationship of subjective and objective performance indicators in subjective cognitive decline. *Psychology & Neuroscience*, *9*(3), 362–378. <https://doi.org/10.1037/pne0000061>

Murari, G., Liang, D. R.-S., Ali, A., Chan, F., Mulder-Heijstra, M., Verhoeff, N. P. L. G., Herrmann, N., Chen, J. J., & Mah, L. (2020). Prefrontal GABA levels correlate with memory in older adults at high risk for Alzheimer’s disease. *Cerebral Cortex Communications*, *1*(1), tgaa022.<https://doi.org/10.1093/texcom/tgaa022>

Nakhla, M. Z., Bangen, K. J., Schiehser, D. M., Roesch, S., & Zlatar, Z. Z. (2023). Greater subjective cognitive decline severity is associated with worse memory performance and lower entorhinal cerebral blood flow in healthy older adults. *Journal of the International Neuropsychological Society*, 1–10. <https://doi.org/10.1017/S1355617723000115>

Nasreddine, Z. S., Phillips, N. A., Bédirian, V., Charbonneau, S., Whitehead, V., Collin, I., Cummings, J. L., & Chertkow, H. (2005). The Montreal Cognitive Assessment, MoCA: a brief screening tool for mild cognitive impairment. *Journal of the American Geriatrics Society*, *53*(4), 695–699. <https://doi.org/10.1111/j.1532-5415.2005.53221.x>

National Heart, Lung, and Blood Institute (2014). *Quality Assessment Tool for Observational Cohort and Cross-Sectional Studies.* <https://www.nhlbi.nih.gov/health-topics/study-quality-assessment-tools>

Nellessen, N., Onur, O. A., Richter, N., Jacobs, H. I. L., Dillen, K. N. H., Reutern, B. von, Langen, K. J., Fink, G. R., & Kukolja, J. (2021). Differential neural structures, intrinsic functional connectivity, and episodic memory in subjective cognitive decline and healthy controls. *Neurobiology of Aging*, *105*, 159–173.<https://doi.org/10.1016/j.neurobiolaging.2021.04.016>

Nguyen, L. A., Haws, K. A., Fitzhugh, M. C., Torre, G. A., Hishaw, G. A., & Alexander, G. E. (2016). Interactive effects of subjective memory complaints and hypertension on learning and memory performance in the elderly. *Neuropsychology, Development, and Cognition*, *23*(2), 154–170. <https://doi.org/10.1080/13825585.2015.1063580>

Nikolai, T., Bezdicek, O., Markova, H., Stepankova, H., Michalec, J., Kopecek, M., Dokoupilova, M., Hort, J., & Vyhnalek, M. (2018). Semantic verbal fluency impairment is detectable in patients with subjective cognitive decline. *Applied Neuropsychology. Adult*, *25*(5), 448–457. <https://doi.org/10.1080/23279095.2017.1326047>

Nobili, F., Frisoni, G. B., Portet, F., Verhey, F., Rodriguez, G., Caroli, A., Touchon, J., Calvini, P., Morbelli, S., de Carli, F., Guerra, U. P., de Pol, L. A., & Visser, P.-J. (2008). Brain SPECT in subtypes of mild cognitive impairment. Findings from the DESCRIPA multicenter study. *Journal of Neurology*, *255*(9), 1344–1353. <https://doi.org/10.1007/s00415-008-0897-4>

Nunes, T., Fragata, I., Ribeiro, F., Palma, T., Maroco, J., Cannas, J., Secca, M., Menezes, C., Carmo, I., Cunha, G., Castelo Branco, M., Guerreiro, M., & de Mendonca, A. (2010). The outcome of elderly patients with cognitive complaints but normal neuropsychological tests. *Journal of Alzheimer’s Disease : JAD*, *19*(1), 137–145.<https://doi.org/10.3233/JAD-2010-1210>

Nutter-Upham, K. E., Saykin, A. J., Rabin, L. A., Roth, R. M., Wishart, H. A., Pare, N., & Flashman, L. A. (2008). Verbal fluency performance in amnestic MCI and older adults with cognitive complaints. *Archives of Clinical Neuropsychology: The Official Journal of the National Academy of Neuropsychologists*, *23*(3), 229–241. <https://doi.org/10.1016/j.acn.2008.01.005>

Oh, Y.-S., Kim, J.-S., Park, J.-W., An, J.-Y., Park, S. K., Shim, Y.-S., Yang, D.-W., & Lee, K.-S. (2016). Arterial stiffness and impaired renal function in patients with Alzheimer’s disease. *Neurological Sciences: Official Journal of the Italian Neurological Society and of the Italian Society of Clinical Neurophysiology*, *37*(3), 451–457. <https://doi.org/10.1007/s10072-015-2434-4>

Oliver, M. D., Morrison, C., Kamal, F., Graham, J., & Dadar, M. (2022). Subjective cognitive decline is a better marker for future cognitive decline in females than in males. *Alzheimer’s Research & Therapy*, *14*(1), 197. <https://doi.org/10.1186/s13195-022-01138-w>

Oltra-Cucarella, J., Ferrer-Cascales, R., Alegret, M., Gasparini, R., Díaz-Ortiz, L. M., Ríos, R., Martínez-Nogueras, Á. L., Onandia, I., Pérez-Vicente, J. A., Cabello-Rodríguez, L., & Sánchez-SanSegundo, M. (2018). Risk of progression to Alzheimer's disease for different neuropsychological Mild Cognitive Impairment subtypes: A hierarchical meta-analysis of longitudinal studies. *Psychology and Aging, 33*(7), 1007–1021. https://doi.org/10.1037/pag0000294

Opdebeeck, C., Yates, J. A., Kudlicka, A., & Martyr, A. (2019). What are subjective cognitive difficulties and do they matter? *Age and Ageing*, *48*(1), 122–127. <https://doi.org/10.1093/ageing/afy148>

Page, M. J., McKenzie, J. E., Bossuyt, P. M., Boutron, I., Hoffmann, T. C., Mulrow, C. D., Shamseer, L., Tetzlaff, J. M., Akl, E. A., Brennan, S. E., Chou, R., Glanville, J., Grimshaw, J. M., Hróbjartsson, A., Lalu, M. M., Li, T., Loder, E. W., Mayo-Wilson, E., McDonald, S., … Moher, D. (2021). The PRISMA 2020 statement: An updated guideline for reporting systematic reviews. *BMJ*, *372*(71). <https://doi.org/10.1136/bmj.n71>

Pappas, C., Small, B. J., Andel, R., Laczo, J., Parizkova, M., Ondrej, L., & Hort, J. (2019). Blood glucose levels may exacerbate executive function deficits in older adults with cognitive impairment. *Journal of Alzheimer’s Disease: JAD*, *67*(1), 81–89. <https://doi.org/10.3233/JAD-180693>

Parizkova, M., Andel, R., Lerch, O., Markova, H., Gazova, I., Vyhnalek, M., Hort, J., & Laczo, J. (2017). Homocysteine and real-space navigation performance among non-demented older adults. *Journal of Alzheimer’s Disease: JAD*, *55*(3), 951–964. <https://doi.org/10.3233/JAD-160667>

Park, S., Lee, J.-H., Lee, J., Cho, Y., Park, H. G., Yoo, Y., Youn, J.-H., Ryu, S.-H., Hwang, J. Y., Kim, J., & Lee, J.-Y. (2019). Interactions between subjective memory complaint and objective cognitive deficit on memory performances. *BMC Geriatrics*, *19*(1), 294. <https://doi.org/10.1186/s12877-019-1322-9>

Pavel A., Matei V., Paun R., & Tudose C. (2022). How “subjective” is subjective cognitive decline? *Psychiatry and Clinical Psychopharmacology*, *32*(4), 299–305. <https://doi.org/10.5152/pcp.2022.22506>

Peng, S., Wang, C., Lin, S., Lee, Y., Lin, Y., Lin, Y., & Wang, P. (2023). Subjective cognitive complaints: Comparing the relation between self-reported versus informant-reported subjective cognitive complaints and cognitive performances in cognitively unimpaired, mild cognitive impairment and populations with dementia. *Journal of Prevention of Alzheimer’s Disease*, *10,* 562-570. <https://doi.org/10.14283/jpad.2023.47>

Perez, V., Garrido-Chaves, R., Zapater-Fajari, M., Pulopulos, M. M., Barbosa, F., Hidalgo, V., & Salvador, A. (2022). Deficits in facial emotional valence processing in older people with subjective memory complaints: Behavioral and electrophysiological evidence. *Psychophysiology*, *59*(4), e13989.<https://doi.org/10.1111/psyp.13989>

Perrotin, A., la Joie, R., de La Sayette, V., Barre, L., Mezenge, F., Mutlu, J., Guilloteau, D., Egret, S., Eustache, F., & Chetelat, G. (2017). Subjective cognitive decline in cognitively normal elders from the community or from a memory clinic: Differential affective and imaging correlates. *Alzheimer’s & Dementia: The Journal of the Alzheimer’s Association*, *13*(5), 550–560. <https://doi.org/10.1016/j.jalz.2016.08.011>

Peter, J., Scheef, L., Abdulkadir, A., Boecker, H., Heneka, M., Wagner, M., Koppara, A., Kloppel, S., Jessen, F., & Alzheimer’s Disease Neuroimaging, I. (2014). Gray matter atrophy pattern in elderly with subjective memory impairment. *Alzheimer’s & Dementia: The Journal of the Alzheimer’s Association*, *10*(1), 99–108. <https://doi.org/10.1016/j.jalz.2013.05.1764>

Petersen, R. C., Doody, R., Kurz, A., Mohs, R. C., Morris, J. C., Rabins, P. V., Ritchie, K., Rossor, M., Thal, L., & Winblad, B. (2001). Current concepts in mild cognitive impairment. *Archives of Neurology*, *58*(12), 1985–1992. <https://doi.org/10.1001/archneur.58.12.1985>

Pietschnig, J., Aigner-Wober, R., Reischenbock, N., Kryspin-Exner, I., Moser, D., Klug, S., Auff, E., Dal-Bianco, P., Pusswald, G., & Lehrner, J. (2016). Facial emotion recognition in patients with subjective cognitive decline and mild cognitive impairment. *International Psychogeriatrics*, *28*(3), 477–485. <https://doi.org/10.1017/S1041610215001520>

Polcher, A., Frommann, I., Koppara, A., Wolfsgruber, S., Jessen, F., & Wagner, M. (2017). Face-name associative recognition deficits in subjective cognitive decline and mild cognitive impairment. *Journal of Alzheimer’s Disease: JAD*, *56*(3), 1185–1196. <https://doi.org/10.3233/JAD-160637>

Pusswald, G., Moser, D., Pfluger, M., Gleiss, A., Auff, E., Stogmann, E., Dal-Bianco, P., & Lehrner, J. (2016). The impact of depressive symptoms on health-related quality of life in patients with subjective cognitive decline, mild cognitive impairment, and Alzheimer’s disease. *International Psychogeriatrics*, *28*(12), 2045–2054. <https://doi.org/10.1017/S1041610216001289>

Rabin, L. A., Chi, S. Y., Wang, C., Fogel, J., Kann, S. J., & Aronov, A. (2014). Prospective memory on a novel clinical task in older adults with mild cognitive impairment and subjective cognitive decline. *Neuropsychological Rehabilitation*, *24*(6), 868–893. <https://doi.org/10.1080/09602011.2014.915855>

Rabin, L. A., Roth, R. M., Isquith, P. K., Wishart, H. A., Nutter-Upham, K. E., Pare, N., Flashman, L. A., & Saykin, A. J. (2006). Self- and informant reports of executive function on the BRIEF-A in MCI and older adults with cognitive complaints. *Archives of Clinical Neuropsychology : The Official Journal of the National Academy of Neuropsychologists*, *21*(7), 721–732. <https://doi.org/10.1016/j.acn.2006.08.004>

Rabin, L. A., Saykin, A. J., Wishart, H. A., Nutter-Upham, K. E., Flashman, L. A., Pare, N., & Santulli, R. B. (2007). The Memory and Aging Telephone Screen: Development and preliminary validation. *Alzheimer’s & Dementia: The Journal of the Alzheimer’s Association*, *3*(2), 109–121. <https://doi.org/10.1016/j.jalz.2007.02.002>

Rabin, L. A., Smart, C. M., Crane, P. K., Amariglio, R. E., Berman, L. M., Boada, M., Buckley, R. F., Chételat, G., Dubois, B., Ellis, K. A., Gifford, K. A., Jefferson, A. L., Jessen, F., Katz, M. J., Lipton, R. B., Luck, T., Maruff, P., Mielke, M. M., Molinuevo, J. L., … Sikkes, S. A. M. (2015). Subjective cognitive decline in older adults: An overview of self-report measures used across 19 international research studies. *Journal of Alzheimer’s Disease*, *48*(S1), S63–S86. <https://doi.org/10.3233/JAD-150154>

Rabin, L. A., Wang, C., Mogle, J. A., Lipton, R. B., Derby, C. A., & Katz, M. J. (2020). An approach to classifying subjective cognitive decline in community‐dwelling elders. *Alzheimer’s & Dementia : Diagnosis, Assessment & Disease Monitoring, 12*(1), e12103–n/a. https://doi.org/10.1002/dad2.12103

Rabin, L., Sikkes, S. A. M., Tommet, D., Jones, R., Crane, P .K., Elbulok-Charcape, M. M., Dubbelman, M. A… & the SCD-I Working Group. (2023). Linking self-perceived cognitive functioning questionnaires using item response theory: The Subjective Cognitive Decline Initiative. *Neuropsychology, 37* (4), 463-499. <https://doi.org/10.1037/neu0000888>

Ramakers, I. H. G. B., Visser, P. J., Bittermann, A. J. N., Ponds, R. W. H. M., van Boxtel, M. P. J., & Verhey, F. R. J. (2009). Characteristics of help-seeking behaviour in subjects with subjective memory complaints at a memory clinic: A case-control study. *International Journal of Geriatric Psychiatry*, *24*(2), 190–196. <https://doi.org/10.1002/gps.2092>

Rami, L., Mollica, M. A., Garcia-Sanchez, C., Saldana, J., Sanchez, B., Sala, I., Valls-Pedret, C., Castellvi, M., Olives, J., & Molinuevo, J. L. (2014). The Subjective Cognitive Decline Questionnaire (SCD-Q): A validation study. *Journal of Alzheimer’s Disease: JAD*, *41*(2), 453–466. <https://doi.org/10.3233/JAD-132027>

Reid, L. M., & MacLullich, A. M. J. (2006). Subjective memory complaints and cognitive impairment in older people. *Dementia and Geriatric Cognitive Disorders*, *22*(5–6), 471–485. <https://doi.org/10.1159/000096295>

Reisberg, B., Ferris, S., de Leon, M., & Crook, T. (1982). The global deterioration scale for assessment of primary degenerative dementia. *The American Journal of Psychiatry*, *139*, 1136–1139. <https://doi.org/10.1176/ajp.139.9.1136>

Reisberg, B., Shulman, M. B., Torossian, C., Leng, L., & Zhu, W. (2010). Outcome over seven years of healthy adults with and without subjective cognitive impairment. *Alzheimer’s & Dementia: The Journal of the Alzheimer’s Association*, *6*(1), 11–24. <https://doi.org/10.1016/j.jalz.2009.10.002>

Rimajova, M., Lenzo, N. P., Wu, J.-S., Bates, K. A., Campbell, A., Dhaliwal, S. S., McCarthy, M., Rodrigues, M., Paton, A., Rowe, C., Foster, J. K., & Martins, R. N. (2008). Fluoro-2-deoxy-D-glucose (FDG)-PET in APOEepsilon4 carriers in the Australian population. *Journal of Alzheimer’s Disease: JAD*, *13*(2), 137–146. <https://doi.org/10.3233/jad-2008-13203>

Rivera-Fernandez, C., Custodio, N., & Soto-Anari, M. (2021). Neuropsychological profile in the preclinical stages of dementia: Principal component analysis approach. *Dementia & Neuropsychologia*, *15*(2), 192–199.<https://doi.org/10.1590/1980-57642021dn15-020006>

Rodda, J., Dannhauser, T., Cutinha, D. J., Shergill, S. S., & Walker, Z. (2011). Subjective cognitive impairment: functional MRI during a divided attention task. *European Psychiatry: The Journal of the Association of European Psychiatrists*, *26*(7), 457–462. <https://doi.org/10.1016/j.eurpsy.2010.07.003>

Rodda, J. E., Dannhauser, T. M., Cutinha, D. J., Shergill, S. S., & Walker, Z. (2009). Subjective cognitive impairment: increased prefrontal cortex activation compared to controls during an encoding task. *International Journal of Geriatric Psychiatry*, *24*(8), 865–874. <https://doi.org/10.1002/gps.2207>

Rosas A.G., Stogmann E., & Lehrner J. (2022). Neuropsychological prediction of dementia using the neuropsychological test battery Vienna—A retrospective study. *Brain Disorders*, *5*. <https://doi.org/10.1016/j.dscb.2021.100028>

Rotenberg Shpigelman, S., Sternberg, S., & Maeir, A. (2019). Beyond memory problems: Multiple obstacles to health and quality of life in older people seeking help for subjective memory complaints. *Disability and Rehabilitation*, *41*(1), 19–25. <https://doi.org/10.1080/09638288.2017.1370729>

Rouch, I., Anterion, C. T., Dauphinot, V., Kerleroux, J., Roche, F., Barthelemy, J. C., & Laurent, B. (2009). Cognitive complaints, neuropsychological performance and affective disorders in elderly community residents. *Disability and Rehabilitation*, *30*(23), 1794–1802. <https://doi.org/10.1080/09638280701667825>

Ryu, S. Y., Kim, A., Kim, S. Y., Park, K. W., Park, K. H., Youn, Y. C., Lee, D. W., Lee, J. Y., Lee, J. H., Jeong, J. H., Choi, S. H., Han, H. J., Kim, S., Na, S., Park, M., Yim, H. W., & Yang, D. W. (2020). Self- and informant-reported cognitive functioning and awareness in subjective cognitive decline, mild cognitive impairment, and very mild Alzheimer disease. *International Journal of Geriatric Psychiatry*, *35*(1), 91–98. <https://doi.org/10.1002/gps.5224>

Sakurai, R., Suzuki, H., Ogawa, S., Kawai, H., Yoshida, H., Hirano, H., Ihara, K., Obuchi, S., & Fujiwara, Y. (2017). Fear of falling, but not gait impairment, predicts subjective memory complaints in cognitively intact older adults. *Geriatrics & Gerontology International*, *17*(7), 1125–1131. <https://doi.org/10.1111/ggi.12829>

Sanchez-Benavides, G., Grau-Rivera, O., Cacciaglia, R., Suarez-Calvet, M., Falcon, C., Minguillon, C., Gramunt, N., Sala-Vila, A., Gispert, J. D., & Molinuevo, J. L. (2018). Distinct cognitive and brain morphological features in healthy subjects unaware of informant-reported cognitive decline. *Journal of Alzheimer’s Disease: JAD*, *65*(1), 181–191. <https://doi.org/10.3233/JAD-180378>

Saunders, N. L. J., & Summers, M. J. (2010). Attention and working memory deficits in mild cognitive impairment. *Journal of Clinical and Experimental Neuropsychology*, *32*(4), 350–357. <https://doi.org/10.1080/13803390903042379>

Seidel, S., Dal-Bianco, P., Pablik, E., Muller, N., Schadenhofer, C., Lamm, C., Klosch, G., Moser, D., Klug, S., Pusswald, G., Auff, E., & Lehrner, J. (2015). Depressive symptoms are the main predictor for subjective sleep quality in patients with mild cognitive impairment–A Controlled Study. *PloS One*, *10*(6), e0128139. <https://doi.org/10.1371/journal.pone.0128139>

Shao, W., Li, X., Zhang, J., Yang, C., Tao, W., Zhang, S., Zhang, Z., & Peng, D. (2019). White matter integrity disruption in the pre-dementia stages of Alzheimer’s disease: from subjective memory impairment to amnestic mild cognitive impairment. *European Journal of Neurology*, *26*(5), 800–807. <https://doi.org/10.1111/ene.13892>

Shirooka, H., Nishiguchi, S., Fukutani, N., Tashiro, Y., Nozaki, Y., & Aoyama, T. (2018). Subjective cognitive decline and fall risk in community-dwelling older adults with or without objective cognitive decline. *Aging Clinical and Experimental Research*, *30*(5), 457–462. <https://doi.org/10.1007/s40520-017-0799-3>

Slavin, M. J., Sachdev, P. S., Kochan, N. A., Woolf, C., Crawford, J. D., Giskes, K., Reppermund, S., Trollor, J. N., Draper, B., Delbaere, K., & Brodaty, H. (2015). Predicting cognitive, functional, and diagnostic change over 4 years using baseline subjective cognitive complaints in the Sydney Memory and Ageing Study. *The American Journal of Geriatric Psychiatry: Official Journal of the American Association for Geriatric Psychiatry*, *23*(9), 906–914. <https://doi.org/10.1016/j.jagp.2014.09.001>

Smart, C. M., Karr, J. E., Areshenkoff, C. N., Rabin, L. A., Hudon, C., Gates, N., Ali, J. I., Arenaza-Urquijo, E. M., Buckley, R. F., Chetelat, G., Hampel, H., Jessen, F., Marchant, N. L., Sikkes, S. A. M., Tales, A., Flier, W. M. van der, & Wesselman, L. (2017). Non-pharmacologic interventions for older adults with subjective cognitive decline: Systematic review, meta-analysis, and preliminary recommendations. *Neuropsychology Review*, *27*(3), 245–257. <https://doi.org/10.1007/S11065-017-9342-8>

Smart, C. M., & Krawitz, A. (2015). The impact of subjective cognitive decline on Iowa Gambling Task performance. *Neuropsychology*, *29*(6), 971–987. <https://doi.org/10.1037/neu0000204>

Smart, C. M., Segalowitz, S. J., Mulligan, B. P., & MacDonald, S. W. S. (2014). Attention capacity and self-report of subjective cognitive decline: a P3 ERP study. *Biological Psychology*, *103*, 144–151. <https://doi.org/10.1016/j.biopsycho.2014.08.016>

Snitz, B. E., Morrow, L. A., Rodriguez, E. G., Huber, K. A., & Saxton, J. A. (2008). Subjective memory complaints and concurrent memory performance in older patients of primary care providers. *Journal of the International Neuropsychological Society*, *14*(6), 1004–1013. <https://doi.org/10.1017/s1355617708081332>

Sohrabi, H. R., Bates, K. A., Rodrigues, M., Taddei, K., Laws, S. M., Lautenschlager, N. T., Dhaliwal, S. S., Johnston, A. N. B., Mackay-Sim, A., Gandy, S., Foster, J. K., & Martins, R. N. (2009). Olfactory dysfunction is associated with subjective memory complaints in community-dwelling elderly individuals. *Journal of Alzheimer’s Disease: JAD*, *17*(1), 135–142. <https://doi.org/10.3233/JAD-2009-1020>

Sohrabi, H. R., Weinborn, M., Laske, C., Bates, K. A., Christensen, D., Taddei, K., Rainey-Smith, S. R., Brown, B. M., Gardener, S. L., Laws, S. M., Martins, G., Burnham, S. C., Bucks, R. S., Reisberg, B., Lautenschlager, N. T., Foster, J., & Martins, R. N. (2019). Subjective memory complaints predict baseline but not future cognitive function over three years: results from the Western Australia Memory Study. *International Psychogeriatrics*, *31*(4), 513–525. <https://doi.org/10.1017/S1041610218001072>

Song, I.-U., Choi, E. K., Oh, J. K., Chung, Y.-A., & Chung, S.-W. (2016). Alteration patterns of brain glucose metabolism: comparisons of healthy controls, subjective memory impairment and mild cognitive impairment. *Acta Radiologica,* *57*(1), 90–97. <https://doi.org/10.1177/0284185114566088>

Song, M., Lee, S. H., Kim, S. Y., & Kang, Y. (2021). Measurement of subjective cognitive decline (SCD) using Korean-Everyday Cognition (K-ECog) as a screening tool: A feasibility study. *Dementia and Neurocognitive Disorders*, *20*(4), 80–88.<https://doi.org/10.12779/dnd.2021.20.4.80>

Steinberg, S. I., Negash, S., Sammel, M. D., Bogner, H., Harel, B. T., Livney, M. G., McCoubrey, H., Wolk, D. A., Kling, M. A., & Arnold, S. E. (2013). Subjective memory complaints, cognitive performance, and psychological factors in healthy older adults. *American Journal of Alzheimer’s Disease and Other Dementias*, *28*(8), 776–783. <https://doi.org/10.1177/1533317513504817>

Tahmasebi, R., Zehetmayer, S., Stogmann, E., & Lehrner, J. (2020). Awareness of olfactory dysfunction in subjective cognitive decline, mild cognitive decline, and Alzheimer’s disease. *Chemosensory Perception*, *13*(1), 59–70. <https://doi.org/10.1007/s12078-019-09267-7>

Tao, W., Sun, J., Li, X., Shao, W., Pei, J., Yang, C., Wang, W., Xu, K., Wang, J., & Zhang, Z. (2020). The anterior-posterior functional connectivity disconnection in the elderly with subjective memory impairment and amnestic mild cognitive impairment. *Current Alzheimer Research*, *17*(4), 373–381. <https://doi.org/10.2174/1567205017666200525015017>

The National Academies: Committee on Psychological Testing, Including Validity Testing, for Social Security Administration Disability Determinations; Board on the Health of Select Populations; Institute of Medicine (2015). Cognitive Tests and Performance Validity Tests. In: *Psychological Testing in the Service of Disability Determination*. National Academies Press (US). <https://www.ncbi.nlm.nih.gov/books/NBK305230/>

Thomas, K. R., Edmonds, E. C., Eppig, J., Salmon, D. P., Bondi, M. W., & Alzheimer’s Disease Neuroimaging, I. (2018). Using neuropsychological process scores to identify subtle cognitive decline and predict progression to mild cognitive impairment. *Journal of Alzheimer’s Disease: JAD*, *64*(1), 195–204. <https://doi.org/10.3233/JAD-180229>

Torrens-Burton, A., Basoudan, N., Bayer, A. J., & Tales, A. (2017). Perception and reality of cognitive function: Information processing speed, perceived memory function, and perceived task difficulty in older adults. *Journal of Alzheimers Disease*, *60*(4), 1601–1609. <https://doi.org/10.3233/jad-170599>

Tyndall, A. V., Longman, R. S., Sajobi, T. T., Parboosingh, J. S., Drogos, L. L., Davenport, M. H., Eskes, G. A., Hogan, D. B., Hill, M. D., & Poulin, M. J. (2020). Genetic risk, vascular function, and subjective cognitive complaints predict objective cognitive function in healthy older adults: Results from the brain in motion study. *Frontiers in Integrative Neuroscience*, *14*, 571683.<https://doi.org/10.3389/fnint.2020.571683>

Valech, N., Tort-Merino, A., Coll-Padrós, N., Olives, J., León, M., Rami, L., & Molinuevo, J. L. (2018). Executive and language subjective cognitive decline complaints discriminate preclinical Alzheimer’s Disease from normal aging. *Journal of Alzheimer’s Disease*, *61*(2), 689–703. <https://doi.org/10.3233/JAD-170627>

Valencia N. & Lehrner J. (2021). Assessing visuo-constructive functions in patients with subjective cognitive decline, mild cognitive impairment and Alzheimer’s disease with the Vienna Visuo-Constructional Test 3.0 (VVT 3.0). *Neuropsychiatrie*, *35*(3), 147–155.<https://doi.org/10.1007/s40211-021-00385-x>

van Rooden, S., van den Berg-Huysmans, A. A., Croll, P. H., Labadie, G., Hayes, J. M., Viviano, R., van der Grond, J., Rombouts, S. A. R. B., & Damoiseaux, J. S. (2018). Subjective cognitive decline is associated with greater white matter hyperintensity volume. *Journal of Alzheimers Disease*, *66*(3), 1283–1294. <https://doi.org/10.3233/JAD-180285>

Verfaillie, S. C. J., Pichet Binette, A., Vachon-Presseau, E., Tabrizi, S., Savard, M., Bellec, P., Ossenkoppele, R., Scheltens, P., van der Flier, W. M., Breitner, J. C. S., Villeneuve, S., & Group, P.-A. R. (2018). Subjective cognitive decline is associated with altered default mode network connectivity in individuals with a family history of Alzheimer’s disease. *Biological Psychiatry. Cognitive Neuroscience and Neuroimaging*, *3*(5), 463–472. <https://doi.org/10.1016/j.bpsc.2017.11.012>

Visser, P. J., Verhey, F., Knol, D. L., Scheltens, P., Wahlund, L.-O., Freund-Levi, Y., Tsolaki, M., Minthon, L., Wallin, A. K., Hampel, H., Burger, K., Pirttila, T., Soininen, H., Rikkert, M. O., Verbeek, M. M., Spiru, L., & Blennow, K. (2009). Prevalence and prognostic value of CSF markers of Alzheimer’s disease pathology in patients with subjective cognitive impairment or mild cognitive impairment in the DESCRIPA study: A prospective cohort study. *The Lancet. Neurology*, *8*(7), 619–627. <https://doi.org/10.1016/S1474-4422(09)70139-5>

Viviano, R. P., Hayes, J. M., Pruitt, P. J., Fernandez, Z. J., van Rooden, S., van der Grond, J., Rombouts, S. A. R. B., & Damoiseaux, J. S. (2019). Aberrant memory system connectivity and working memory performance in subjective cognitive decline. *NeuroImage*, *185*, 556–564. <https://doi.org/10.1016/j.neuroimage.2018.10.015>

Vogel, A., Bruus, A., & Waldemar, G. (2022.). Developing a Danish version of the LASSI-L test—Reliability and predictive value in patients with mild cognitive impairment, mild dementia due to AD and subjective cognitive decline. *Aging, Neuropsychology, and Cognition*. <https://doi.org/10.1080/13825585.2022.2133076>

Wang, P. J., Saykin, A. J., Flashman, L. A., Wishart, H. A., Rabin, L. A., Santulli, R. B., McHugh, T. L., MacDonald, J. W., & Mamourian, A. C. (2006). Regionally specific atrophy of the corpus callosum in AD, MCI and cognitive complaints. *Neurobiology of Aging*, *27*(11), 1613–1617. <https://doi.org/https://doi.org/10.1016/j.neurobiolaging.2005.09.035>

Wang, Q., Chen, B., Zhong, X., Zhou, H., Zhang, M., Mai, N., Wu, Z., Huang, X., Haehner, A., Chen, X., Auber, L. A., Peng, Q., Hummel, T., & Ning, Y. (2021). Olfactory dysfunction is already present with subjective cognitive Decline and deepens with disease severity in the Alzheimer’s disease spectrum. *Journal of Alzheimer’s Disease : JAD*, *79*(2), 585–595.<https://doi.org/10.3233/JAD-201168>

Wang, S., Rao, J., Yue, Y., Xue, C., Hu, G., Qi, W., ... Chen, J. (2021). Altered frequency-dependent brain activation and white matter integrity associated with cognition in characterizing preclinical Alzheimer’s disease stages. *Frontiers in Human Neuroscience, 15*, 625232. <https://doi.org/10.3389/fnhum.2021.625232>

Wang, X., Li, T., Zhang, H., Sun, T., Xiong, L., Zhang, Y., Sun, Z., Yu, X., & Wang, H. (2019). Validation of the Chinese version of the memory binding test for distinguishing amnestic mild cognitive impairment from cognitively normal elderly individuals. *International Psychogeriatrics*, *31*(12), 1721–1730. <https://doi.org/10.1017/S1041610219001649>

Wang, X., Wang, Z., Hu, H., Qu, Y., Wang, M., Shen, X., Xu, W., Dong, Q., Tan, L., & JT, Y. (2021). Association of subjective cognitive decline with risk of cognitive impairment and dementia: A systematic review and meta-analysis of prospective longitudinal studies. *The Journal of Prevention of Alzheimer’s Disease*, *8*(3), 277-285. <https://doi.org/10.14283/JPAD.2021.27>

Wereszczynski, M., & Niedzwienska, A. (2021). Dementia-free older adults with subjective cognitive impairment show lower mood and no deficits of spontaneous memory retrieval. *International Journal of Aging & Human Development*, *95*(3), 372–394. <https://doi.org/10.1177/00914150211066561>

Wolfsgruber, S., Kleineidam, L., Guski, J., Polcher, A., Frommann, I., Roeske, S., Spruth, E. J., Franke, C., Priller, J., Kilimann, I., Teipel, S., Buerger, K., Janowitz, D., Laske, C., Buchmann, M., Peters, O., Menne, F., Fuentes Casan, M., Wiltfang, J., … DELCODE Study Group. (2020). Minor neuropsychological deficits in patients with subjective cognitive decline. *Neurology*, *95*(9), e1134–e1143.<https://doi.org/10.1212/WNL.0000000000010142>

Yang, L., Yan, Y., Li, Y., Hu, X., Lu, J., Chan, P., Yan, T., & Han, Y. (2019). Frequency-dependent changes in fractional amplitude of low-frequency oscillations in Alzheimer’s disease: A resting-state fMRI study. *Brain Imaging and Behavior*, *14*, 2187-2201. <https://doi.org/10.1007/s11682-019-00169-6>

Yen-Hsuan, H., Ching-Feng, H., Min-Chien, T., & Mau-Sun, H. (2015). Prospective memory in subjective cognitive decline. *Alzheimer Disease and Associated Disorders*, *29*(3), 229–235. <https://doi.org/10.1097/WAD.0000000000000060>

Yoon, B., Choi, S. H., Jeong, J. H., Park, K. W., Kim, E. J., Hwang, J., Jang, J. W., Kim, H. J., Hong, J. Y., Lee, J. M., Kang, J. H., & Yoon, S. J. (2020). Balance and mobility performance along the Alzheimer’s disease spectrum. *Journal of Alzheimer’s Disease*, *73*(2), 633–644. <https://doi.org/10.3233/JAD-190601>

Yuan Q., Liang X., Xue C., Qi W., Chen S., Song Y., Wu H., Zhang X., Xiao C., & Chen J. (2022). Altered anterior cingulate cortex subregional connectivity associated with cognitions for distinguishing the spectrum of pre-clinical Alzheimer’s disease. *Frontiers in Aging Neuroscience*, *14*. <https://doi.org/10.3389/fnagi.2022.1035746>

Zhang Z., Cui L., Huang Y., Chen Y., Li Y., & Guo Q. (2021). Changes of regional neural activity homogeneity in preclinical Alzheimer’s disease: Compensation and dysfunction. *Frontiers in Neuroscience*, *15*, 646414.<https://doi.org/10.3389/fnins.2021.646414>

Zheng, Z., Zhao, X., Cui, X., Liu, X., Zhu, X., Jiang, Y., & Li, J. (2023). Subtle pathophysiological changes in working memory-related potentials and intrinsic theta power in community-dwelling older adults with subjective cognitive decline. *Innovation in Aging*, *7*(2). <https://doi.org/10.1093/geroni/igad004>

Zlatar, Z. Z., Muniz, M., Galasko, D., & Salmon, D. P. (2018). Subjective cognitive decline correlates with depression symptoms and not with concurrent objective cognition in a clinic-based sample of older adults. *The Journals of Gerontology. Series B, Psychological sciences and social sciences*, *73*(7), 1198–1202. <https://doi.org/10.1093/geronb/gbw207>

Zlatar, Z. Z., Tarraf, W., Gonzalez, K. A., Vasquez, P. M., Marquine, M. J., Lipton, R. B., Gallo, L. C., Khambaty, T., Zeng, D., Youngblood, M. E., Estrella, M. L., Isasi, C. R., Daviglus, M., & Gonzalez, H. M. (2022). Subjective cognitive decline and objective cognition among diverse U.S. Hispanics/Latinos: Results from the Study of Latinos-Investigation of Neurocognitive Aging (SOL-INCA). *Alzheimer’s & Dementia : The Journal of the Alzheimer’s Association*, *18*(1), 43–52. <https://doi.org/10.1002/alz.12381>
